# Pleiotropy or linkage? Their relative contributions to the genetic correlation of quantitative traits and detection by multitrait GWA studies

**DOI:** 10.1093/genetics/iyab159

**Published:** 2021-09-25

**Authors:** Jobran Chebib, Frédéric Guillaume

**Affiliations:** 1 Department of Evolutionary Biology and Environmental Studies, University of Zürich, 8057 Zürich, Switzerland; 2 Institute of Evolutionary Biology, University of Edinburgh, Edinburgh EH9 3FL, UK; 3 Organismal and Evolutionary Biology Research Program, University of Helsinki, 00014 Helsinki, Finland

**Keywords:** genetic architecture, correlational selection, multivariate, forward-time simulation, SNP, spurious pleiotropy, trait mapping

## Abstract

Genetic correlations between traits may cause correlated responses to selection. Previous models described the conditions under which genetic correlations are expected to be maintained. Selection, mutation, and migration are all proposed to affect genetic correlations, regardless of whether the underlying genetic architecture consists of pleiotropic or tightly linked loci affecting the traits. Here, we investigate the conditions under which pleiotropy and linkage have different effects on the genetic correlations between traits by explicitly modeling multiple genetic architectures to look at the effects of selection strength, degree of correlational selection, mutation rate, mutational variance, recombination rate, and migration rate. We show that at mutation-selection(-migration) balance, mutation rates differentially affect the equilibrium levels of genetic correlation when architectures are composed of pairs of physically linked loci compared to architectures of pleiotropic loci. Even when there is perfect linkage (no recombination within pairs of linked loci), a lower genetic correlation is maintained than with pleiotropy, with a lower mutation rate leading to a larger decrease. These results imply that the detection of causal loci in multitrait association studies will be affected by the type of underlying architectures, whereby pleiotropic variants are more likely to be underlying multiple detected associations. We also confirm that tighter linkage between nonpleiotropic causal loci maintains higher genetic correlations at the traits and leads to a greater proportion of false positives in association analyses.

## Introduction 

Both pleiotropy and linkage disequilibrium (LD) create genetic correlations between traits so that traits do not vary independently of one another (Wright 1977; Arnold 1992; Walsh and Blows 2009). Under natural selection, strong genetic correlation can prevent a combination of traits from reaching their respective optimum trait values (Falconer and Mackay 1996). Likewise, under artificial selection, it can constrain breeders from improving one trait due to undesired changes in another, and in medical gene-targeted therapy treatments, it can cause adverse side-effects (Wright 1977; Parkes *et al.* 2013; Visscher *et al.* 2017; Wei and Nielsen 2019). Pleiotropy can cause genetic correlation because one gene’s product (*e.g.*, an enzyme or a transcription factor) has more than one target and therefore affects more than one trait or because one gene’s product belongs to a metabolic pathway that has more than one downstream effect (Hodgkin 1998; Stearns 2010; Wagner and Zhang 2011). LD may be the result of a set of loci in close physical proximity on a chromosome that makes a set of alleles at those loci less likely to be split up by recombination and therefore more likely to get passed on together from one generation to the next. But other mechanisms leading to the transmission of one combination of alleles at separate loci over another combination can also generate LD and create genetic correlations between traits that those loci affect (*e.g.*, drift, migration, assortative mating; Falconer and Mackay 1996).

The evolutionary dynamics of pleiotropic versus linked loci in creating genetic correlations are expected to be similar under the assumptions of complete linkage (no recombination) between pairs of loci each affecting a different trait, weak stabilizing selection, weak mutational effects, and large mutation rates (Lande 1984). Correlational selection is when selection favors correlated values at phenotypic traits, which should translate into correlated genetic effects at the underlying loci affecting the traits. This has been shown to be the case in models attempting to approximate the level of genetic variance and covariance maintained at mutation-selection balance at pleiotropic loci affecting polygenic traits (Lande 1980; Turelli 1985; Zhang and Hill 2003; Chantepie and Chevin 2020). For the case of tight linkage between pairs of loci affecting separate traits, Lande (1984) suggested that it “is nearly equivalent to” pleiotropic loci affecting both traits. Therefore, the genetic correlation maintained under correlational selection by tight linkage between nonpleiotropic loci should in principle approximate that of pleiotropic loci predicted by previous multivariate quantitative genetics models (Lande 1980; Zhang and Hill 2003). However, the conditions under which this statement is correct have not been fully elucidated. Yet, the fact that the same equilibrium genetic correlation under correlational selection has been obtained under different theoretical assumptions about the rate and effect size of mutations by Lande (1984) and Zhang and Hill (2003) tends to suggest that Lande’s statement may hold generally (see also Chantepie and Chevin 2020, for a recent reanalysis).

Under assumptions of weak mutational effects and relatively high mutation rates (such that mutational variance is lower than the standing genetic variance of the trait, settings known as the Gaussian mutation regime; Lande 1975), Lande (1984) determined that the maximum genetic correlation due to pleiotropy or linkage may be almost as large as the strength of correlational selection, which can be calculated from the genetic covariance and the genetic variances of the traits, respectively, as:

(1) $$\mathrm{genetic}\;\mathrm{covariance}\;(b)=\frac{\rho_{\omega }\omega^{2}\mu \alpha^{2}}{2c},$$

(2) $$\mathrm{genetic}\;\mathrm{variance}\;(c)=\sqrt[{}]{(1+\sqrt{1-\rho_{\omega }^{2}})\omega^{2}\frac{\mu \alpha^{2}}{2}},$$

where $\rho_{\omega }$ is the strength of correlational selection acting between the traits, $\omega^{2}$ is the strength of stabilizing selection (with lower values representing stronger selection), *μ* is the haploid per-locus mutation rate, and $\alpha^{2}$ is the per-locus mutational variance. The model is for an infinite population. If there are equal mutation variances and selection strengths among traits then the genetic correlation is calculated as:

(3) $$\mathrm{genetic}\;\mathrm{correlation}=\frac{b}{c}=\frac{\rho_{\omega }}{1+\sqrt{1-\rho_{\omega }^{2}}}.$$

From Equations (1 and 2), we see that the genetic covariance depends on both the strength of correlational selection between traits and selection on each trait, as well as on the mutational inputs (mutation rate and mutational variance) of the genes affecting those traits. Yet, from Equation (3), the resulting genetic correlation among traits is independent of the genetic architecture of the traits because the influences of the mutational input on the variance and the covariance cancel out exactly.

Interestingly, the same result was obtained by Zhang and Hill (2003) under the assumptions of the house-of-cards mutation regime (most of the genetic variance of the trait is contributed by few mutations of large effect, see Turelli 1984; Bulmer 1989; Johnson and Barton 2005; Walsh and Lynch 2018). Equations (1–3) assume that the mutations at pleiotropic loci have uncorrelated effect on the traits, *i.e.*, there is no mutational correlation ($\rho_{\mu }=0$). Chantepie and Chevin (2020) recently showed that the equivalence between the Gaussian and house-of-cards regimes holds even when $\rho_{\mu }\neq 0$. They further derived the equilibrium G-matrix under the Gaussian regime at mutation–selection–drift balance, which will be useful in this study for generating expectations under Gaussian assumptions. For that same case of a multivariate, noncorrelated Gaussian distribution of mutational effects at pleiotropic loci, Lande goes on further to state that the case of complete linkage between pairs of loci affecting different traits is “equivalent to a lesser number of loci with pleiotropic effects,” suggesting that equations (1–3) also apply to pairs of linked loci. However, the difference between pleiotropic and linked nonpleiotropic loci has not been quantified nor has the scaling of the genetic variance and covariance been examined.

One key difference between pleiotropic and nonpleiotropic loci is that pleiotropic loci require only one mutation to affect multiple traits and build up genetic covariation, while linked nonpleiotropic loci require as many mutations as the number of traits, with each locus affecting a separate trait. The mutation rate may thus play an important role in distinguishing the two types of genetic architectures. This is because a given mutation at a pair of linked loci can only affect one trait. Therefore, linked loci may not be strictly equivalent to pleiotropic loci because of their lack of mutational covariation. In contrast, mutations at pleiotropic loci provide the opportunity for combinations of effects in all directions of phenotype space to match patterns of correlational selection better than mutations at linked loci. However, Lande’s (1984) derivations assume a similar multivariate Gaussian distribution of allele effects for the pleiotropic and nonpleiotropic loci.

Additionally, levels of trait genetic covariation can be influenced by other evolutionary processes that affect allele frequencies, and the covariation of allelic values in a population [*e.g.*, migration (Guillaume and Whitlock 2007), drift (Griswold *et al.* 2007; Chantepie and Chevin 2020), inbreeding (Lande 1984), and phenotypic plasticity (Draghi and Whitlock 2012)]. Migration affects genetic covariation because when it is sufficiently high (relative to selection in the focal population), then combinations of alleles coming from a source population will also be maintained in the focal population. This can lead to higher genetic covariation between traits in the focal populations, whether the combinations of alleles immigrating are (more likely to be) correlated in their effects on those traits or not (Guillaume and Whitlock 2007). Migration may also have different effects depending on whether the genetic architecture is pleiotropic or made up of linked loci, but this has not been explored either. Recombination can also reduce genetic correlations between traits by breaking up associations between alleles at linked loci, but the same cannot occur with a pleiotropic locus. These considerations therefore suggest that linkage and pleiotropy can have different effects on genetic variance and covariances depending on mutation, recombination, and selection regimes, but this comparison was not fully explored in any previous model.

Finally, knowing how linked loci affect genetic correlations among traits is important when utilizing a genome-wide association studies (GWAS) to identify causal genetic variants underlying one or more traits. GWAS use the rapid increase in genomic sequencing to find correlations between traits and genotypes, and their success is dependent on the effect sizes of the loci and the distinction between phenotypes. GWAS have had success in associating genetic variants with traits of interest, which have allowed researchers to find the molecular underpinnings of trait change (Visscher *et al.* 2017). Moving from one trait to two or more trait associations can lead to the discovery of pleiotropic loci (Saltz *et al.* 2017). One GWAS using 1094 traits and 14,459 genes, found that 44% of genes were “pleiotropic,” but this was determined by assigning genetic variants to the closest gene and even to both flanking genes when the genetic variant was intergenic (Chesmore *et al.* 2018). This conflates linkage and pleiotropy, and the chain of causality (Platt *et al.* 2010). Another study found 81% of associated genes and 60% of associated single-nucleotide polymorphisms (SNPs) were pleiotropic, but they could not rule out SNPs associated with traits due to LD (Watanabe *et al.* 2019). Unfortunately, determining whether genetic variant associations and trait correlations are actually the result of pleiotropy or linkage is difficult since they often map to large regions of genomes, or are in intergenic regions and do not associate with the closest genes (Flint and Mackay 2009; Zhu *et al.* 2016; Peichel and Marques 2017; Visscher *et al.* 2017). Distinguishing between the two types of genetic architectures is important for understanding the underlying molecular functions of the traits, and determining how the traits may be differently affected by selection (Lynch *et al.* 1998; Barrett and Hoekstra 2011; Saltz *et al.* 2017). This is salient at a time when an increasing number of traits of interest (*e.g.*, human diseases) appear to be affected by loci that affect other traits, and especially when targeted gene therapy clinical trials are more widespread than ever (Edelstein *et al.* 2007; Cai *et al.* 2016; Pickrell *et al.* 2016; Visscher and Yang 2016; Chesmore *et al.* 2018; Ginn *et al.* 2018). There are potentially negative implications for gene therapy because repairing a gene underlying one disease might increase the risk for another disease. For example, some genetic variants that are associated with greater risk of Ankylosing spondylitis are also associated with less risk of rheumatoid arthritis, and so “repairing” a specific allele would have undesired side-effects in this case (Parkes *et al.* 2013; Gratten and Visscher 2016).

Here, we are interested in the conditions under which pleiotropic loci behave similarly or differently to tightly linked loci affecting different traits, with respect to their effects on genetic correlations between the traits. We first derive a mathematical expression for the variance–covariance genetic structure of a pair of traits affected by a pair of nonpleiotropic loci. We then use computer simulations to investigate whether the effect of evolutionary forces on the genetic correlation between traits is dependent on the type of genetic architecture (pleiotropic or linked loci), and mutation regime (Gaussian and house-of-cards). We focus on the relative contributions of selection, mutation, recombination, and migration to the build-up of genetic correlation between traits having different genetic architectures. We show that unless mutation rates are high as in the Gaussian regime, genetic architectures with tight linkage between loci maintain lower equilibrium genetic correlations than pleiotropic architectures. Even when mutation rates are high, other evolutionary forces affecting equilibrium levels of genetic correlation still show a difference between architectures but to a much lesser extent. In addition, we simulate genomic SNP data sets using the different architectures and show that map distances between causative and noncausative quantitative trait locus (QTL) affect false-positive proportions in GWA analyses.

## Model

We seek to express the variance–covariance matrix of two quantitative traits as a function of the mutational input at two diploid nonpleiotropic linked loci, which is considered to be equivalent to one pleiotropic locus in Lande (1984). We use the same mutational parameters as in equations (1–2): Mutational effects, *a*, at a locus are drawn from a Gaussian distribution $N(0,\alpha^{2})$ with mean 0 and variance $\alpha^{2}$ and are added to the existing allelic value at rate *μ*. To better express the difference between pleiotropic and nonpleiotropic loci, we introduce a second parameter *θ* such that the column vector of mutation effects on traits 1 and 2 can be expressed as:

(4) $$a={\left(a\;\text{cos}(\theta ),a\;\text{sin}(\theta )\right)}^{T},$$

where *θ* represents the direction of the mutational vector in the two-trait plane, and ^*T*^ denotes transpose. The angle *θ* is uniformly distributed between 0 and 2π for pleiotropic loci, while it can only take values [0,π/2,π,3π/2] for a pair of nonpleiotropic loci. The distribution of effects at a pleiotropic locus is a bivariate Gaussian $N_{2}(0,M)$ with mean zero, a diagonal variance–covariance matrix $M=\alpha^{2}I$, as in Lande (1984), and **I** is the two-dimensional identity matrix. In contrast, the mutational input for two nonpleiotropic loci is the weighted sum of two normal distributions along trait 1 or trait 2 with probability μ(1−μ) contributed by separate mutations in either locus and of a bivariate distribution with probability $\mu^{2}$ contributed by two mutations arising in both loci at the same time. The full distribution of mutation effects at a pair of two haploid loci (x), with $x={\left(x_{1},x_{2}\right)}^{T}$, *x*_1_ affecting trait 1 and *x*_2_ trait 2, can be written as:

(5) $$f(x)=\frac{\mu (1-\mu )N(0,\alpha^{2}){\left(\text{cos}(\theta ),\;\text{sin}(\theta )\right)}^{T}+\mu^{2}N_{2}(0,M)}{1-{\left(1-\mu \right)}^{2}}.$$

Because of the negligible contribution of double mutations for μ≪1, f(x) is basically a cross set along both axes of phenotype space, far from a bivariate Gaussian as assumed by Lande:

(6) $$f(x)=\frac{\mu (1-\mu )}{1-{\left(1-\mu \right)}^{2}}\cdot \{\begin{array}{cc} {\left(0,\;E[a]\right)}^{T} & \;\text{for}\;\theta =\left[\pi /2\;\text{or}\;3\pi /2\right]\\ {\left(E[a],\;0\right)}^{T} & \;\text{for}\;\theta =\left[0\;\text{or}\;\pi \right] \end{array},$$

with E[a] the expected average mutation effect with $a∼N(0,\alpha^{2})$.

Stabilizing selection on the two traits is represented by a bivariate Gaussian surface with variance $\omega^{2}$ and correlation $\rho_{\omega }$, giving the selection matrix:

(7) $$\Omega =\omega^{2}\left(\begin{array}{cc} 1 & \rho_{\omega }\\ \rho_{\omega } & 1 \end{array}\right).$$

The G-matrix (genetic variance–covariance matrix) at mutation–selection balance for a single haploid pair of fully linked loci can be found using the house-of-cards approximation for an infinite population sitting at the trait optimum under stabilizing selection (Bulmer 1989):

(8) $$G=2\;\mu \frac{aa^{T}}{a^{T}\cdot \Omega^{-1}\cdot a}.$$

With **a** as in (4) and Ω as in (7), we find:

(9) $$G=2\;\mu \;\omega^{2}\left(\frac{1-\rho_{\omega }^{2}}{1-\rho_{\omega }\;2\;\text{cos}(\theta )\;\text{sin}(\theta )}\right)\left(\begin{array}{cc} \;\text{cos}\;{\left(\theta \right)}^{2} & \;\text{cos}(\theta )\;\text{sin}(\theta )\\ \;\text{cos}(\theta )\;\text{sin}(\theta ) & \;\text{sin}\;{\left(\theta \right)}^{2} \end{array}\right).$$

**Figure 1:**
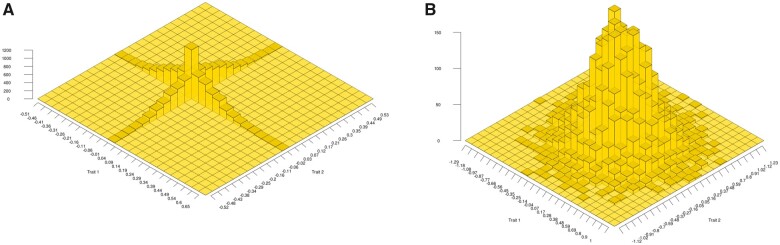
Distributions of mutational effects on two traits, Trait 1 (*x*-axis) and Trait 2 (*y*-axis). Graph (A) illustrates the distribution obtained from equation (5) for 10,000 nonpleiotropic mutations at a pair of linked loci with $\alpha^{2}=0.1$ and $\mu =10^{-5}$. Graph (B) illustrates the distribution of pleiotropic mutations with the same characteristics as in (A) obtained from a bivariate normal distribution.

From (9) and the distribution of *θ*, it is clear that mutations at nonpleiotropic loci cannot generate genetic covariance between the traits because the matrix part of (9) becomes $\left(\begin{array}{cc} 1 & 0\\ 0 & 1 \end{array}\right)$ for θ∈[0,π/2,π,3π/2]. The same conclusion is reached from (5) for continuous effects at the two loci. The distribution of mutation effects at two linked loci is principally concentrated on the two trait axes, as illustrated in Figure 1A. Therefore, nonpleiotropic mutations do not directly contribute to genetic covariance at the two traits unless the mutation rate is very high. The same is true for multiple pairs of loci when the pairs are unlinked, assuming linkage equilibrium among pairs, as in Lande (1984). Nevertheless, covariance should principally build-up from linkage disequilibria within pairs, caused by correlational selection on the two traits. Note that the pleiotropic case is fully covered in Lande (1980, 1984); Zhang and Hill (2003); Chantepie and Chevin (2020).

## Simulations

**Figure 2:**
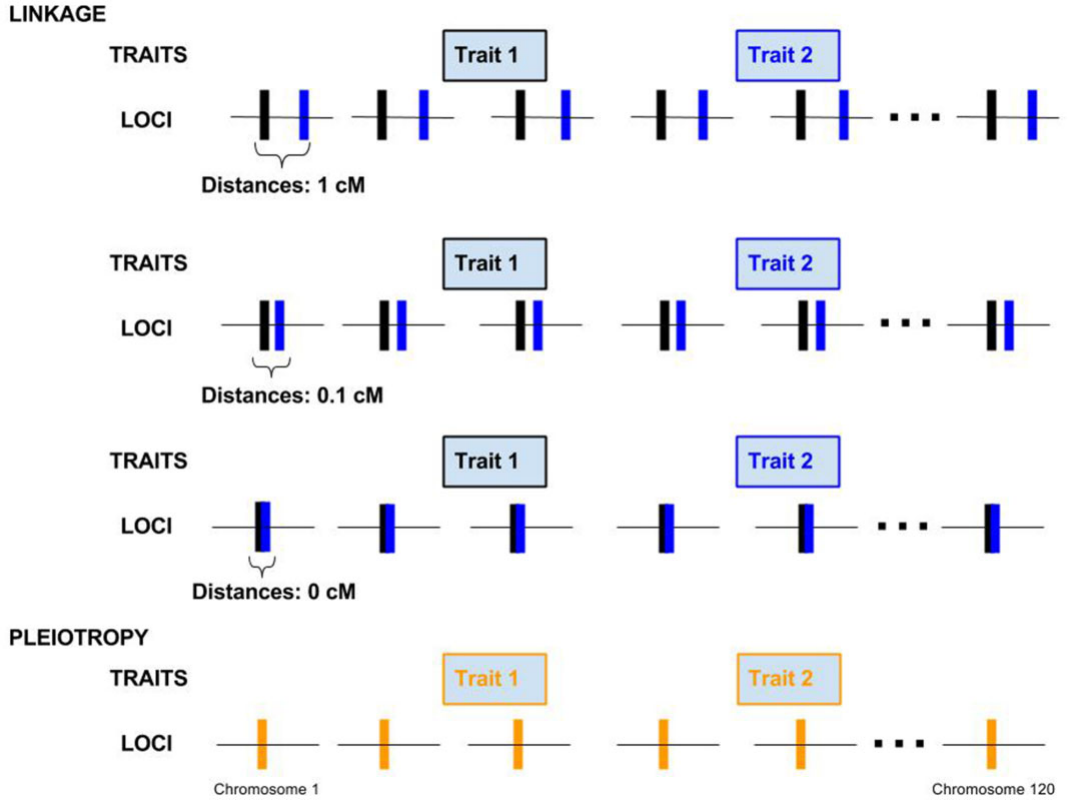
Four genetic architectures showing the distribution of loci on 120 linkage groups. In the case of nonpleiotropic pairs of loci affecting the two different traits on each linkage group are either 1, 0.1, or 0 cM apart. In the case of the pleiotropic architecture, each locus on each chromosome affects both traits.

We built individual-based simulations to understand the dynamics of the genetic correlation at linked nonpleiotropic loci and compare with equilibrium values at pleiotropic loci, incorporating different levels of LD stemming from physical linkage, selection, and migration. We modeled four different genetic architectures in a modified version of the individual-based, forward-in-time, population genetics simulation software Nemo (Guillaume and Rougemont 2006; Chebib and Guillaume 2017). Nemo was modified to allow individual nonpleiotropic loci to affect different quantitative traits. To compare how pleiotropy and linkage differentially affect the genetic correlation between traits, we modeled a set of 120 pairs of linked, nonpleiotropic loci, and a set of 120 pleiotropic loci affecting the two traits. The traits were thus polygenic, as expected for many phenotypic traits in plants and animals (Mackay 2001; Boyle *et al.* 2017; Sella and Barton 2019; Barghi *et al.* 2020). We also ran a set of simulations with 60 loci affecting each trait as a comparison. We varied the recombination distance between the two nonpleiotropic loci of each pair with distances 0, 0.1, or 1 cM (Figure 2). Pairs were unlinked to other pairs. The pleiotropic loci were also unlinked to each other. The recombination rates chosen represent no recombination between linked loci, as well as an average and an extreme value of recombination at “hotspots” in the human genome, respectively (Myers *et al.* 2006). All loci had additive effects on the traits.

Unless otherwise specified, each simulation was run with 5000 initially mono-morphic, diploid individuals (variation is gradually introduced through mutations) for 50,000 generations achieving mutation–selection–drift(-migration) balance in order to observe general patterns of genetic correlation. Individuals were hermaphrodites mating at random within a population, with nonoverlapping generations. Phenotypes were calculated for each of the two traits modeled by summing the allelic values of all loci affecting each trait. Gaussian stabilizing selection was applied and determined the survival probability of juveniles, whose fitness was calculated as $w=\text{exp}\;\left[-\frac{1}{2}\left({\left(z-\theta \right)}^{T}\cdot \Omega^{-1}\cdot (z-\theta )\right)\right]$, where **z** is the individual phenotype vector (initialized to the optimum values), *θ* is the vector of local optimal trait values (set to 10 for both traits in the focal population), and Ω is the selection variance–covariance matrix (Equation 7). We explored the effect of the strength of stabilizing selection by setting $\omega^{2}=$ 50, or 100, and the strength of correlational selection but setting $\rho_{\omega }$ = 0.5 or 0.9. The strength of selection scales inversely with $\omega^{2}$ where a value of 100 corresponds to weak (but nontrivial) selection. In contrast, a value of $\rho_{\omega }=0.9$ corresponds to strong correlational selection between traits (Lande 1984; Turelli 1985).

To examine the effects of mutational input on genetic correlation between traits, we contrasted two types of loci: loci with small effects and large mutation rates against loci of large but rare effects. These two contrasted architectures correspond to the assumptions of the Gaussian and house-of-cards regime, respectively (see Turelli 1984). In both cases, mutation effects are drawn from a normal distribution with mean zero and variance $\alpha^{2}$ at rate *μ* for each haploid locus. Pleiotropic mutation effects are drawn from a bivariate normal distribution with mean and covariance equal to zero, and per-trait variance of $\alpha^{2}$. To model mutational variation under the Gaussian regime, we set the mutation effect size $\alpha^{2}=0.001$ and $\mu =10^{-3}$ such that $\mu \gg \alpha^{2}/\omega^{2}$ (Turelli 1984, 1985). The house-of-cards regime corresponds to the condition $\mu \ll \alpha^{2}/\omega^{2}$ (Latter 1960; Bulmer 1972; Bulmer *et al.* 1980; Turelli 1984, 1985). We thus set $\alpha^{2}=0.1$ and $\mu =10^{-5}$ for that case. The Gaussian regime ensures that mutational variation is lower than the standing variation at the quantitative loci so that the equilibrium distribution of allelic effects at each locus remains approximately normal (Lande 1975). In contrast, under the house-of-cards regime, the distribution of allelic effects at each locus is much more leptokurtic than Gaussian because almost all the variance is contributed by rare alleles of large effect (Turelli 1984; Johnson and Barton 2005). The house-of-cards assumptions seem more plausible because a mutational variance $\alpha^{2}$ larger than the standing variance at each locus is needed for more realistic mutation rates $\mu \ll 10^{-3}$ to maintain sufficient genetic variance at mutation–selection balance, as argued by Turelli (1984) (see also Bulmer 1989; Bürger 2000; Johnson and Barton 2005).

To study the continuity of effects between both model assumptions, we used all combinations of parameter values for *μ* in {0.001, 0.0001, 0.00001} and $\alpha^{2}$ in {0.001, 0.1}. Mutational effects were then added to the existing allelic values (continuum-of-alleles model; Crow and Kimura 1964). All loci were assumed to have equal mutational variances and mutation rates. For simplicity, no environmental effects on the traits were included. Another set of simulations was run with reduced recombination between pairs of nonpleiotropic, fully linked loci. The pairs were set 0.001, 0.1, or 1 cM apart on a single chromosome to understand the effect of LD between pairs on the build-up of genetic correlation.

To examine the effects of migration from a source population on genetic correlation between traits, additional sets of simulations were run with unidirectional migration from a second population (as in an island-mainland model with each population consisting of 5000 individuals) with backward migration rates (*m*) of 0.1, 0.01, and 0.001. The backward migration rate represents the average proportion of new individuals in the focal population whose parent is from the source population. The local optimum values for the two traits in the source population were set at $\theta =\left[\sqrt{50},\sqrt{50}\right]$ (10 units distance from the focal population’s local optimum). Both focal and source populations had weak stabilizing selection with a strength of $\omega^{2}=100$, the focal population had no correlational selection between the two traits and the source population had a correlational selection of $\rho_{\omega }=$ 0 or 0.9. Fifty replicate simulations were run for each set of parameter values and statistics were averaged over replicates. Averages were also compared against analytical expectations laid out by Lande (1984) and reproduced here in equations 1–3.

### Mapping pleiotropic and nonpleiotropic loci with GWA analysis

To elucidate the differential effects of pleiotropy and linkage on the detection of true causal genetic variants in association studies, we performed genome-wide association analyses (GWAAs) on a set of additional simulations of neutral genetic loci linked to the causative QTL. The neutral loci were diallelic with a per-allele mutation rate of $\mu =10^{-6}$. We placed 1000 neutral loci around each pleiotropic locus or nonpleiotropic pair of completely linked loci, thus simulating an additional 120,000 potentially polymorphic neutral loci linked to the additive, multiallelic quantitative loci described before. The causative QTL was set in the center of their linkage groups (chromosomes). Each chromosome’s map length was set to 0.1 cM, with neutral loci set equidistantly on each, resulting in a recombination rate of $10^{-6}$ between adjacent loci. We simulated a single Wright–Fisher population with *N_e_* = 5000 for 50,000 generations and saved the complete neutral SNP and selected QTL sequences for a sample of 1000 individuals at the end of each of 10 simulation replicates. The polymorphic SNP and QTL were extracted from those sequences and used for the GWA analyses. Univariate GWAAs were performed on each trait independently with custom R-scripts using simple linear regression. Multivariate GWAAs were performed on both traits with a multivariate linear mixed model implemented in the software *gemma* (v0.98.1) (Zhou and Stephens 2014). Results were obtained from command “gemma -bfile sim_bed_file -maf 0.001 -lmm 2 -n 1 2 -k sim_GRM -o output” with the kinship matrix (genetic relatedness matrix) obtained from the command: “gemma -bfile sim_bed_file -maf 0.001 -gk -o sim_GRM.” We used the *P*-values obtained from likelihood-ratio tests. To determine the significance of associations of neutral SNPs with trait variation, we transformed the *P*-values to *q*-values with the *q-value* R package (v2.14.1) (Storey *et al.* 2019) using a significance threshold (FDR) of α=0.1 (Storey and Tibshirani 2003). We considered a segregating QTL discovered when at least one SNP was found significant on the same chromosome, within 0.05 cM on each side of the QTL. The probability of detecting spurious pleiotropy can then be assessed by comparing the discovery rates (DRs) of nonpleiotropic versus pleiotropic QTL in a multivariate GWAA. DRs were computed as the proportion of polymorphic QTL found in the vicinity of neutral SNPs that were significantly associated with one or both phenotypes.

Finally, to better understand the differential effects of pleiotropy and linkage on the detection of true causal genetic variants, we modeled QTL as biallelic loci with the same allelic effect sizes as set in the continuous case. Mutations then only change the sign of the allelic effect at the loci. This way, causative SNPs, or quantitative trait nucleotides (QTNs), can be mapped to trait variation similarly to marker SNPs. Significance was determined with custom-made R-scripts performing linear regressions for each segregating SNP on each trait separately, and adjusting *P*-values for multiple comparisons following Benjamini and Hochberg (1995) with an FDR set at α=0.1.

## Simulation results

### Effects of genetic architecture on genetic correlation at mutation–selection balance

**Figure 3:**
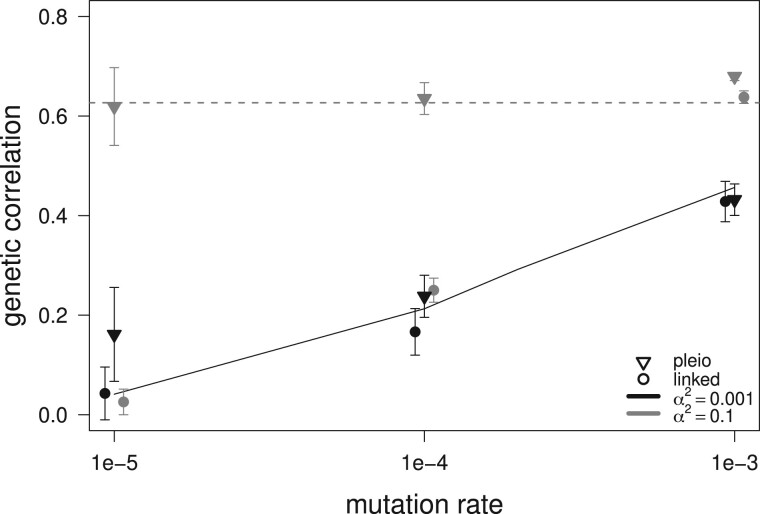
Average genetic correlation between traits 1 and 2 at mutation–selection balance after 50,000 generations of stabilizing selection in one population with 5000 randomly mating hermaphrodites. Average values over 30 replicates are given for simulations with 120 pairs of fully linked nonpleiotropic loci (circles) and 120 pleiotropic loci (triangles). Error bars show one standard deviation. Two sets of mutational parameters were used: $\alpha^{2}=0.001$ (black) and $\alpha^{2}=0.1$ (dark gray). The Gaussian regime ($\alpha^{2}=0.001$) is represented on the right-hand side where μ=1e-3, while the HoC regime ($\alpha^{2}=0.1$) is on the left-hand side where μ=1e-5. The dashed line represents Lande’s (1984) expectations for pairs of linked loci [equation (3)], while the black line is the Gaussian expectation from Chantepie and Chevin (2020). Simulation parameters are *N *=* *5000, $\omega^{2}=100,\;\rho_{\omega }=0.9$.

By generation 50,000, when mutation–selection balance equilibrium is reached, the simulations with pleiotropic loci under the house-of-cards regime maintain a higher average genetic correlation than pleiotropic loci under the Gaussian regime, and higher than simulations with linked nonpleiotropic loci under either regime (Figure 3). The simulations under the Gaussian regime are well approximated by the Gaussian expectations of Chantepie and Chevin (2020). The Gaussian approximation also shows a good match with our house-of-cards simulations when the mutation rate is low (μ ≤ 0.0001) (Figure 3). The mutation-independent expectation for the equilibrium genetic correlation (equation 3) is reached by our pleiotropic architecture under the house-of-cards regime for all mutation rates and for the nonpleiotropic linked pairs only for the highest mutation rate (μ=0.001). Therefore, linked nonpleiotropic loci behave like pleiotropic loci when the mutation rate is high: when $\mu \;\geq \;10^{-4}$ for mutations of small effects ($\alpha^{2}=0.001$, Gaussian regime), and $\mu \;\geq \;10^{-3}$ for mutations of large effects ($\alpha^{2}=0.1$, house-of-cards, hereafter HoC). Overall, we see a tendency for the genetic correlation to increase with the mutation rate for both the pleiotropic and nonpleiotropic loci as predicted by the Gaussian model of Chantepie and Chevin (2020) (see Figure 3). Additional simulations with 60 instead of 120 loci per trait show an average small reduction of the genetic correlation with a reduction of the number of loci (Supplementary Figure S1).

**Figure 4:**
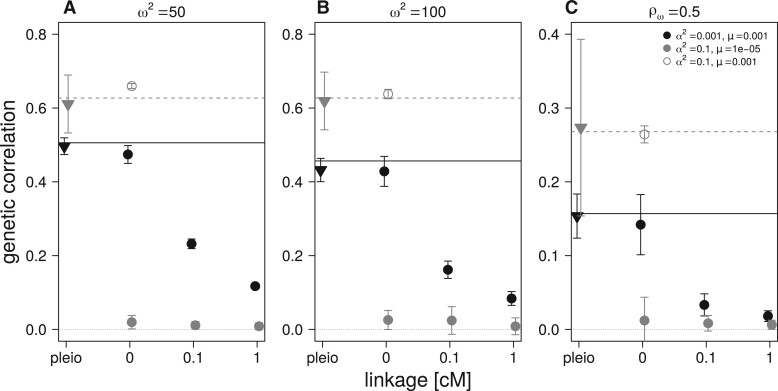
Effect of linkage distance within pairs of nonpleiotropic loci on average genetic correlation after 50,000 generations of correlated, stabilizing selection. Default simulation parameter values are in panel (B): *N *=* *5000, $\omega^{2}=100,\;\rho_{\omega }=0.9$. Stabilizing selection is stronger in panel (A) with $\omega^{2}=50$. Correlational selection is weaker in panel (C) with $\rho_{\omega }=0.5$. Symbols and mutation parameters in inset and as in Figure 3.

**Figure 5:**
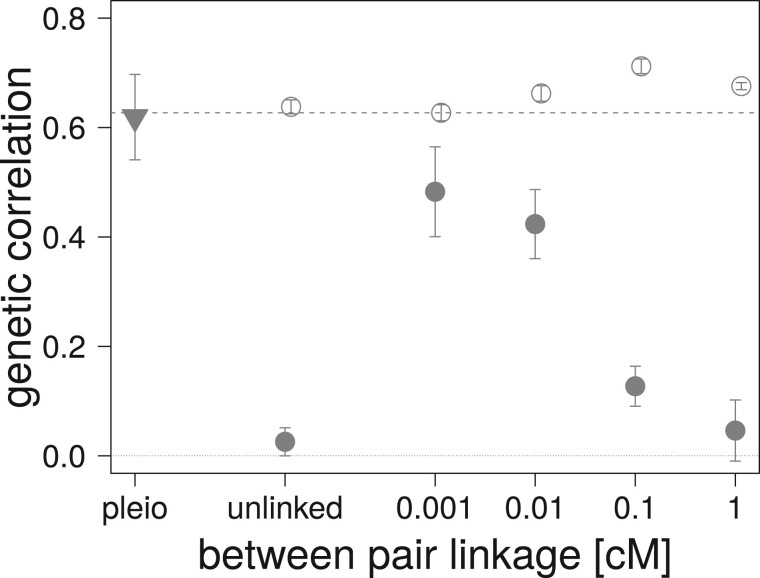
Effect of linkage between pairs of fully linked nonpleiotropic loci on average genetic correlation after 50,000 generations of correlated, stabilizing selection in the HoC regime. Error bars correspond to one standard deviation over 30 replicates. *N *=* *5000, $\omega^{2}=100,\;\rho_{\omega }=0.9,\;\alpha^{2}=0.1$, and $\mu =10^{-5}$ (filled symbols), or $\mu =10^{-3}$ (open circles). The 120 pairs of nonpleiotropic loci were placed on a single chromosome at map distance shown on the *x*-axis. Pleiotropic loci are depicted with an inverse triangle. The dashed line represents Lande’s (1984) expectation.

For the two sets of mutational parameters, under Gaussian and HoC assumptions, the increase in recombination (increased map distance in cM) within the pairs of linked nonpleiotropic loci rapidly decreases the equilibrium genetic correlation between the traits, as expected (Figure 4). Stronger selection (obtained with $\omega^{2}=50$) increases the Gaussian expectation and the observed equilibrium value of both the pleiotropic and completely linked loci in the Gaussian regime, but did not affect the equilibrium reached in the HoC regime (Figure 4A). A similar pattern is observed with selection for a lower phenotypic correlation ($\rho_{\omega }=0.5$) (Figure 4C). At low mutation rates ($\mu =10^{-5}$, HoC), virtually no genetic correlation is observed between the traits affected by pairs of nonpleiotropic loci in all conditions, while pleiotropic loci maintained high genetic correlation in all cases (Figure 4, A–C). In contrast, simulations with a high mutation rate ($\mu =10^{-3}$) and a large mutational effect size ($\alpha^{2}=0.1$, HoC) always reach the level of genetic correlation between the two traits predicted by equation (3) (see open circles in Figure 4). Finally, increased linkage between the pairs of nonpleiotropic loci plays a similar role as increasing the allelic mutation rate; it increases the equilibrium genetic correlation between the trait even when the mutation rate is low ($\mu =10^{-5}$, HoC) (Figure 5).

### Effects of migration on genetic correlation

**Figure 6:**
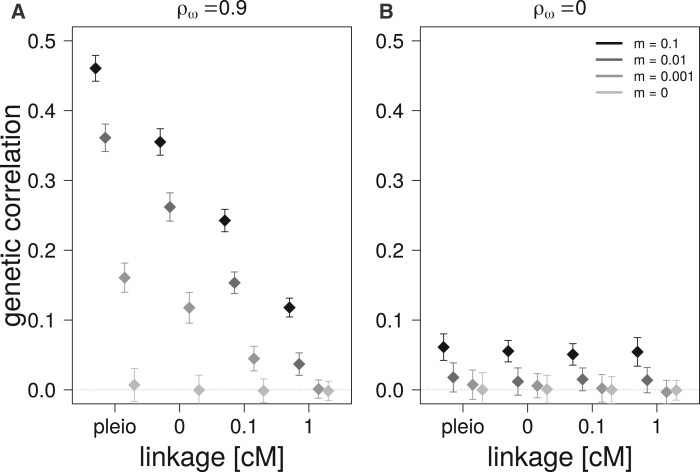
Effect of migration on the average genetic correlations in the focal populations (and their standard deviations) after 10,000 generations of migration from a source population with different migration rates (*m*) for four different genetic architectures. (A) Migration from a source population with correlational selection between traits ($\rho_{\omega }=0.9$). (B) Migration from a source population without correlational selection between traits ($\rho_{\omega }=0$).

A higher migration rate from a source population, whose traits are under correlational selection, leads to higher genetic correlations in the focal population regardless of the genetic architecture (Figure 6A). The effect of migration increases with tighter linkage and is highest with pleiotropic architecture. This effect on genetic correlation is still observed when there is no correlational selection on the traits in the source population, but to a largely reduced degree (Figure 6B).

### Effect of genetic architecture on QTL DRs in a GWAA

In the HoC regime, when mapping neutral SNPs to trait variation, the pleiotropic architecture yields more significantly associated SNPs than the nonpleiotropic architecture, approximately 95 and 70, respectively, out of about 18,000 segregating SNPs (Table 1). The significant SNPs are also in higher LD with pleiotropic than nonpleiotropic segregating QTL. The genetic correlation between the traits does not affect the number of mapped SNPs (*e.g.*, when correlational selection is decreased from 0.9 to 0.06, see Table 1). The difference in number of mapped SNPs is significant between nonpleiotropic and pleiotropic architectures (Welch *t*-test: *P *=* *0.023 for $\rho_{\omega }=0.9$; *P *=* *0.0085 for $\rho_{\omega }=0.06$). The QTL DR of multivariate GWAA is higher for pleiotropic loci (57%) than for nonpleiotropic loci (∼40%) for a similar strength of correlational stabilizing selection ($\rho_{\omega }=0.9$). The difference in DRs shrinks when the same genetic correlation is reached by pleiotropic and nonpleiotropic loci ($G_{\mathrm{cor}}\approx$0.03, with $\rho_{\omega }=0.06$ for pleiotropic loci, see Table 1). The differences in DR are not significant here.

**Table 1:** Summary of the multivariate analyses of neutral SNP associations with the two traits under correlational selection

	$\alpha^{2}$	*μ*	DR${}_{\mathrm{pleio}.}$	*r* ^2^	*G_cor_*	SNP${}_{\mathrm{pleio}.}$	#QTL
Linkage							
* Gauss.*	0.001	10^-3^	0.001 (n.a.)	0.076 (n.a.)	0.419 (0.014)	0.1 (n.a.)	120.0 (0.0)
* *—	0.1	10^-3^	0.002 (n.a.)	0.004 (n.a.)	0.640 (0.003)	0.2 (n.a.)	120.0 (0.0)
* *—	0.001	10^-5^	0.208 (0.015)	0.157 (0.013)	0.032 (0.010)	54.6 (4.8)	90.8 (1.2)
* HoC*	0.1	10^-5^	0.398 (0.035)	0.192 (0.025)	0.032 (0.015)	70.0 (4.3)	73.1 (0.9)
Pleiotropy							
* Gauss.*	0.001	10^-^^3^	0.007 (0.003)	0.039 (0.009)	0.434 (0.014)	1.7 (1.0)	120.0 (0.0)
* *—	0.1	10^-^^3^	0.012 (0.009)	0.010 (0.001)	0.681 (0.004)	1.8 (1.3)	120.0 (0.0)
* *—	0.001	10^-5^	0.260 (0.019)	0.246 (0.016)	0.083 (0.021)	86.1 (8.9)	93.3 (1.7)
* HoC* ${}^{\left(a\right)}$	0.1	10^-5^	0.387 (0.032)	0.268 (0.020)	0.035 (0.035)	94.6 (6.9)	78.8 (1.9)
* HoC* ${}^{\left(b\right)}$	0.1	10^-5^	0.574 (0.037)	0.220 (0.011)	0.610 (0.027)	92.5 (7.7)	68.2 (1.4)

The pleiotropic QTL discovery rate (DR${}_{\mathrm{pleio}.}$) is the proportion of segregating QTL (#QTL) that are significantly associated with an SNP within 0.05 cM. The average LD of the significant SNPs with the closest QTL is evaluated with multiallelic *r*^2^. The significantly associated SNPs (SNP${}_{\mathrm{pleio}.}$) are associated with both traits. *G_cor_* is the mean genetic correlation of the two quantitative traits. All values are means and standard errors (in brackets) for 10 replicates of each simulation assessed at generation 50,000. The GWAAs were performed on samples of 1000 individuals. The mutation parameters of the QTL are in the first three columns and simulation parameters are similar to those in Figure 3. See *Methods* for details about the neutral markers.

${}^{\left(a\right)}\;\rho_{\omega }=0.06$ ; ${}^{\left(b\right)}\;\rho_{\omega }=0.9$; n.a.: the significant SNPs were found in a single replicate.

Compared to the HoC regime, the multivariate GWAA on neutral SNPs in the Gaussian regime had less power to detect significant associations, as shown by the lower number of significant SNPs, their LDs with QTLs, and the QTL DR (<1%) (Table 1). Moreover, only a single replicate out of 10 yielded significant SNP associations in the nonpleiotropic simulations, despite higher genetic correlations than in the HoC regime (0.4 *vs* 0.03, respectively, see Table 1). Therefore, the mutation regime, and especially the mutation rate, was the main factor affecting the significance level of the GWAA and QTL DRs. For a similar mutation rate, significance remains higher for alleles of large effect at the QTL as in the HoC regime ($\alpha^{2}=0.1$) than for alleles of small effect ($\alpha^{2}=0.001$; see Table 1).

Univariate GWAAs on neutral SNPs are less prone than multivariate GWAA to detect marker SNPs associated with trait variation (see number of significant SNPs in Tables 1 and 2). Here, pleiotropic loci are SNPs detected in two univariate GWAA performed independently on traits 1 and 2. Almost no SNPs map to two traits in two independent univariate GWAA unless the QTL are pleiotropic or in the HoC regime (see Table 2). Therefore, the spurious pleiotropy of the neutral markers is very low when compared to the multivariate GWAA. When mapping SNPs associated with nonpleiotropic linked QTL, the false pleiotropy DR of the QTL is 3.5% on average in the HoC regime, and 0% in the Gaussian regime. The true pleiotropy DRs of pleiotropic loci are higher, reaching 4.9% and 0.1% in the HoC and Gaussian regimes, respectively. It is also higher when the genetic correlation between the traits increases from 0.035 to 0.61 in the HoC regime (see *G_cor_* in Table 1) but is lower when the QTL effect size is decreased to 0.001 (see Table 2). The average univariate QTL DRs obtained when mapping a single trait at a time are 23.9% and 0.01% in the HoC and Gaussian regimes, respectively (Table 2). Mutation rates and effect sizes of the QTL played the same role as with multivariate GWAA on both SNP significance and QTL DRs.

**Table 2:** Summary of the univariate analyses of neutral SNP independent associations with each trait

	$\alpha^{2}$	*μ*	DR${}_{\mathrm{pleio}.}$	DR_*single*_	*r* ^2^	SNP${}_{\mathrm{sig}.}$	SNP${}_{\mathrm{pleio}.}$
Linkage							
* Gauss.*	0.001	10^-3^	0.0(*n.a.*)	0.001(0.001)	0.038(0.012)	0.1(0.1)	0.0(*n.a.*)
* *—	0.1	10^-3^	0.0(*n.a.*)	4e−04(*n.a.*)	0.003(*n.a.*)	0.1(*n.a.*)	0.0(*n.a.*)
* *—	0.001	10^-5^	0.009(0.004)	0.105(0.009)	0.242(0.022)	28.9(2.2)	0.0(*n.a.*)
* HoC*	0.1	10^-5^	0.035(0.005)	0.239(0.015)	0.316(0.022)	41.1(2.4)	0.8(0.6)
Pleiotropy							
* Gauss.*	0.001	10^-3^	0.001(0.001)	0.007(0.002)	0.030(0.007)	1.5(0.4)	0.3(*n.a.*)
* *—	0.1	10^-3^	0.001(0.001)	0.007(0.004)	0.025(0.007)	1.1(0.6)	0.2(*n.a.*)
* *—	0.001	10^-5^	0.019(0.004)	0.122(0.008)	0.238(0.016)	37.4(2.6)	2.4(0.9)
* HoC* ${}^{\left(a\right)}$	0.1	10^-5^	0.049(0.008)	0.191(0.015)	0.256(0.020)	44.5(3.5)	6.5(1.6)
* HoC* ${}^{\left(b\right)}$	0.1	10^-5^	0.075(0.006)	0.208(0.019)	0.261(0.022)	39.7(4.5)	14.1(3.5)

The pleiotropy discovery rate (DR${}_{\mathrm{pleio}.}$) is the proportion of segregating QTL detected simultaneously in two independent univariate GWAAs by mapping an SNP within 0.05 cM of the QTL. The QTL discovery rate (DR_*single*_) is the average proportion of QTL discovered by mapping an SNP within 0.05 cM in either one or the other GWAA. LD between the significant SNP and the associated QTL is a multiallelic *r*^2^. The number of SNPs significantly associated with one of the two traits (SNP${}_{\mathrm{sig}.}$) is averaged over the two univariate GWAAs. The number of neutral SNPs that were found to be significant in two GWAAs (SNP${}_{\mathrm{pleio}.}$) represents spurious pleiotropy. The genetic correlation, number of segregating QTL, and simulation details are as in Table 1.

${}^{\left(a\right)}\;\rho_{\omega }=0.06$ ; ${}^{\left(b\right)}\;\rho_{\omega }=0.9$; n.a.: significant SNP in zero or a single replicate.

Finally, the mapping of causative SNPs, or QTNs in univariate GWAAs, shows rates of associations of nonpleiotropic QTNs with unaffected traits (*i.e.*, false-positive rates, FPRs) of 6.5% and 7.4% in the Gaussian and HoC regimes, respectively (Table 3). The rates increase with either increasing the allele effect size to 0.1 in the Gaussian regime or the mutation rate to $10^{-3}$ in the HoC regime. The FPR then reaches up to 9.4% with an increase in the genetic correlation of the traits (Table 3). Spurious pleiotropy of nonpleiotropic QTNs is highest for alleles of small effect with a high mutation rate (FPR = 18.5%, Table 3). In comparison, the pleiotropic QTNs also show lower number of mapped QTNs with a lower mutation rate. The false-negative rate (FNR) is thus higher in the HoC than in the Gaussian regime (Table 3). Those results quantitatively strongly depend on the sample size of the analyses, as shown in Table S4. Increasing the sample size decreases the FNR and increases the FPR as the statistical power of the analysis increases, but the qualitative difference between the Gaussian and the HoC regimes remains.

**Table 3:** Summary of the univariate analyses of causative QTN associations with the two traits for linked pairs of nonpleiotropic and pleiotropic biallelic QTL

							QTN	
	$\alpha^{2}$	*μ*		*r* ^2^	*G_cor_*	*N_sign_*	*N_pleio_*	*N_segr_*
Linkage			*FPR*					
* Gauss.*	0.001	10^-3^	0.065 (0.002)	0.016(0.001)	−4e−04 (0.003)	194.6 (1.0)	12.6 (0.5)	240.0 (0.0)
* *—	0.1	10^-3^	0.094 (0.004)	0.024(0.001)	0.206 (0.003)	177.0 (1.4)	16.8 (0.8)	240.0 (0.0)
* *—	0.001	10^-5^	0.185 (0.006)	0.378(0.013)	0.002 (0.008)	115.0 (1.0)	21.4 (0.8)	188.2 (6.2)
* HoC*	0.1	10^-5^	0.074 (0.006)	0.344(0.046)	0.100 (0.015)	82.6 (1.1)	6.1 (0.5)	149.6 (8.7)
Pleiotropy			*FNR*					
* Gauss.*	0.001	10^-3^	0.700 (0.010)	–(–)	−3e−04 (0.003)	95.1 (0.7)	36.0 (1.2)	120.0 (0.0)
* *—${}^{\left(a\right)}$	0.1	10^-3^	0.584 (0.010)	–(–)	0.189 (0.003)	98.8 (0.7)	49.9 (1.3)	120.0 (0.0)
* *—${}^{\left(b\right)}$	0.001	10^-5^	0.770 (0.007)	–(–)	0.219 (0.013)	71.1 (0.9)	24.1 (0.7)	105.2 (3.6)
* HoC* ${}^{\left(b\right)}$	0.1	10^-5^	0.824 (0.006)	–(–)	0.230 (0.012)	62.7 (0.9)	17.8 (0.6)	100.8 (4.0)

The pleiotropy FPR or spurious pleiotropy is the proportion of nonpleiotropic QTNs significantly associated with both traits in two independent univariate GWAAs. The FNR is the proportion of segregating pleiotropic QTN not associated with the two traits in two independent GWAAs. The mean *r*^2^ measures LD within pairs of nonpleiotropic QTL bearing at least one significant QTN. The sample size is 1000 individuals. The strength of correlational selection is $\rho_{\omega }=0.9$ unless specified otherwise. Other simulation parameters are as presented before.

${}^{\left(a\right)}\;\rho_{\omega }=0.85$ ; ${}^{\left(b\right)}\;\rho_{\omega }=0.01$.

## Discussion

The main expectation under the assumption of weak selection and strong correlational selection is that populations with a genetic architecture consisting of unlinked pairs of two completely linked loci (0-cM distance) should maintain similar equilibrium levels of genetic correlation as a genetic architecture consisting of a lesser number of unlinked pleiotropic loci (Lande 1984). Furthermore, it is expected that pleiotropic loci should yield similar level of genetic correlation at mutation–selection balance irrespective of the mutation regime, assuming mutational effects are not correlated (Lande 1980; Zhang and Hill 2003). Contrary to those expectations, our analysis shows that the genetic architecture and especially the mutation regime determines the genetic correlation of the traits under correlational selection. We show that linked, nonpleiotropic loci yield similar genetic correlations as pleiotropic loci when mutation rates are high as in the Gaussian mutation regime but not when mutations are rare as in the house-of-cards regime. On the other hand, pleiotropic loci reach the deterministic genetic correlation expected under mutation–selection (Lande 1980; Zhang and Hill 2003) for the house-of-cards regime only. Under a Gaussian regime, pleiotropic and nonpleiotropic loci better match the mutation–selection–drift equilibrium of Chantepie and Chevin (2020). Here, a high rate of mutation allows for multiple mutations in tightly linked pairs of QTL to accumulate and maintain levels of genetic covariance near to that of pleiotropic QTL. The same is true for loci of larger effect as in the HoC regime when the per-locus mutation rate is increased. It is, nevertheless, believed that the HoC regime better fits empirical estimates of per-trait standing genetic variance and trait heritability (Turelli 1984, 1985).

Empirical estimations of mutation rates from varied species like bacteria and humans suggest that *per-nucleotide* mutation rates are in the order of $10^{-8}$ to $10^{-9}$ (Nachman and Crowell 2000; Ford *et al.* 2011; Keightley *et al.* 2015; Lindsay *et al.* 2019). If a polygenic locus consists of hundreds or thousands of nucleotides, as in the case of many QTLs, then per-locus mutation rates may be as high as $10^{-5}$ or more, but the larger the locus the higher the chance of recombination between within-locus variants that are contributing to genetic correlation. Also, only a fraction of those mutations will have detectable functional effects. There only are a handful of examples of large loci with suppressed recombination such as a 16-kb region of chromosome IV of threespine stickleback fish and a 155-kb region of chromosome 17 in *t*-haplotype mice (Bullard *et al.* 1992; Herrmann and Bauer 2012; Jones *et al.* 2012). This leads us to believe that with empirically estimated levels of mutation and recombination, strong genetic correlation between traits is more likely to be maintained if there is an underlying pleiotropic architecture affecting them than will be maintained by tight linkage. Nevertheless, as nonpleiotropic loci become more tightly linked on the genetic map, higher genetic covariation can be maintained by correlational selection favoring LD among multiple pairs of nonpleiotropic loci at short recombination distances.

Our main result of a dependency of the equilibrium correlation on the mutation regime was not anticipated because the same mutation-invariant expectation of equilibrium genetic correlation (equation 3) was found under the Gaussian (Lande 1984) and house-of-cards (Zhang and Hill 2003) regimes. However, the recent mutation–selection–drift model of Chantepie and Chevin (2020) provides accurate equilibrium values for loci in the Gaussian regime when taking drift into account. This shows that although our population was relatively large, with $N_{e}<5000$ (it is less than *N* because some self-fertilization is allowed by the mating system), small deviations caused by drift may have a large effect on the orientation of the G-matrix at equilibrium, and thus on the resulting genetic correlation among the traits. As shown in Chantepie and Chevin (2020), the main effect of drift is to bring the G-matrix closer to the variance–covariance matrix of the mutation effects, the M-matrix. In our case, the M-matrix is diagonal, without mutational covariance terms and thus without mutation-induced genetic correlation at the traits. This results in a weakening of the equilibrium genetic correlation among the traits at mutation–selection–drift balance. As expected, stronger selection brings the Gaussian expectation and our simulation results closer to the mutation-invariant equilibrium correlation [Figure 4A, see also Figure 2 in Chantepie and Chevin (2020)].

The equilibrium genetic correlation maintained at nonpleiotropic loci strongly depends on the mutation rate, the number of loci, and the linkage between them. As argued in the Model section, the distribution of mutational effects on the two traits affected by a pair of nonpleiotropic loci is far from a bivariate normal distribution. This represents a stark departure from Lande’s (1980, 1984) assumptions and is the reason why our simulations strongly disagree with the theoretical predictions, unless the mutation rate is high. Indeed, as shown by equation (5), the bivariate normality of the distribution of mutational effects at two loci increases with the square of the mutation rate. This means that with many mutations per locus, it is increasingly likely to generate variation at the two traits simultaneously. It is that variation that is then picked up by correlational selection when mutations increase the traits’ genetic covariance in a direction favored by selection. The number of loci acts in a similar way by increasing the mutation target size for the genetic correlation. In addition, at mutation–selection–recombination balance, Turelli and Barton (1990) (see also Hastings 1989) for the house-of-cards regime and Lande (1975) for the Gaussian regime, have shown that the covariance at a locus pair depends on the product of mutation rates, inverse recombination rate, and selection strength (house-of-cards regime) or allele effects (Gaussian regime). The total covariance being the sum of the covariance at all pairs of loci, it is thus proportional to the number of loci multiplied by the per-locus mutation rate, nμ (*i.e.*, the genomic mutation rate). Therefore, we can generally expect that the total genetic covariance between traits affected by different nonpleiotropic loci increases with nμ, the effect size of the mutations $\alpha^{2}$, and the physical linkage within and between pairs of loci, as shown in our simulations (Supplementary Tables S1–S3).

When simulating different lengths of the genetic map holding the QTL, we have also shown that with tighter linkage *between* pairs of nonpleiotropic linked loci, favored mutations need not affect two loci within the same pair to allow correlational selection to keep them in LD and maintain higher genetic correlation at the traits. LD between pairs can thus do the job. This suggests that high genetic correlations can be maintained under strong correlational selection when many causative loci are clustered within small recombination distances throughout the genome. Our simulations suggest a distance smaller than about 0.01 cM (about 10 kb or less is needed) for loci under the house-of-cards regime and weak selection (or about 100 kb in the Gaussian regime, see Supplementary Figure S2). That distance will increase with the strength of correlational selection, the mutation rate, and the effect size of the mutations, for a given correlation. Therefore, we can expect that selection for phenotypic correlation among polygenic traits can efficiently maintain appreciable levels of genetic correlation even though the causative loci have small mutation rates.

### The impact of pleiotropy and linkage on maintaining different genetic correlations in association studies

When GWA analyses are employed to detect shared genetic influences (pleiotropy or linkage) on multiple traits of interest, they are dependent upon detecting combinations of effect sizes of genetic variants associated with those traits (Hill and Zhang 2012a, 2012b; Chung *et al.* 2014; Visscher and Yang 2016). The success or failure of this endeavor is directly connected to the ability to detect loci with associations to each trait and the strength of genetic correlation between traits (Wei *et al.* 2014; Pickrell *et al.* 2016; Chesmore *et al.* 2018; Verbanck *et al.* 2018). The proportion of genes associated with two or more phenotypes in the human GWAS catalog has been estimated recently to range from 40% (Pickrell *et al.* 2016) to 60% (Watanabe *et al.* 2019), covering between 800 Mbs (Jordan *et al.* 2019; Shikov *et al.* 2020) and 1600 Mbs (Watanabe *et al.* 2019) of the human genome. These rather large differences among studies stem from differences in source data (from 43 to over 550 GWAS), from the extent of correction for correlation among phenotypic traits, from different definitions of trait functional domains, and from various ways of correcting for LD among variants. More stringent analyses still show that pleiotropy is widespread in the human genome (Jordan *et al.* 2019; Shikov *et al.* 2020). But it is difficult to determine if this is truly representative of the prevalence of pleiotropy because QTLs are often mapped to loci that can encompass thousands of nucleotides (and more than one gene) and informative SNPs with significant effect sizes are assigned to the closest genes with annotated phenotypes (Chesmore *et al.* 2018; Liu *et al.* 2019; Cai *et al.* 2020). Conflating intergenic SNPs with nearby pleiotropic genes (or loci) can distort the prevalence of pleiotropy and reduce the ability to distinguish pleiotropy from physical linkage.

When considering noncausal variants, our results show that significant associations are more prevalent for SNPs linked to pleiotropic than to nonpleiotropic loci, even for a similar genetic correlation maintained by both types of architecture at equilibrium. That difference can be explained by the fact that two linked nonpleiotropic loci carry two different sets of alleles, contrary to a single pleiotropic locus carrying only a single set. An SNP associated with a pair of nonpleiotropic loci must thus be in LD with both sets to be categorized as pleiotropic in a GWAA. The LD of a marker SNP with two QTL within a pair is reduced when compared to a single pleiotropic QTL because the two physically linked QTL are by far not in perfect LD even if perfectly physically linked (see Supplementary Table S1). This is seen in their lower genic covariance (*i.e.*, within gamete allele effect covariance) when compared to pleiotropic loci (Supplementary Table S1). Therefore, the power to detect SNPs associated with two traits is decreased for nonpleiotropic relative to pleiotropic architectures. It results in lower FPRs, and thus less spurious pleiotropy. This is especially the case when multiple univariate GWAAs are utilized to map variants to multiple traits, showing less spurious pleiotropy than multivariate GWAA [see also Fernandes *et al.* (2021), for comparisons of uni- and multivariate GWAA]. That advantage of the univariate approach is counterbalanced by its reduced power to discover truly pleiotropic QTL associated with significant SNPs compared to multivariate GWAA.

We also show that the mutational regime strongly affects the SNP and QTL DRs. As expected, QTL with alleles of larger effect sizes as in the HoC regime yield a higher number of significantly associated variants, in both multi- and univariate GWAA. Under the HoC regime, the distribution of allelic effect per locus has a much higher kurtosis (see Supplementary Table S1), meaning that fewer alleles explain most of the additive variation compared to the Gaussian regime. The kurtosis of the distribution strongly decreases with higher mutation rates, and the accompanying larger number of segregating alleles per QTL (*e.g.*, from <3 to ∼100 alleles, see Supplementary Table S1). With higher mutation rates, the contribution of each allele to trait variation is consequently reduced and so is the LD between markers and QTL, decreasing DRs. In comparison, the DR reduction in the Gaussian regime is small for a 100 times reduction of effect sizes relative to the HoC regime. This shows that variation in allele effect sizes at linked loci affects GWAA FPR, as expected (Platt *et al.* 2010). It also suggests that the reduction of pleiotropy (false) DRs in nonpleiotropic architectures is likely accentuated by reductions of the effect size of the QTL, making it more likely for GWAS to detect true pleiotropic relationships across a range of effect sizes and variation of the total mutation rate of the QTL underlying the genetic correlation among traits.

The way genetic parameters affect spurious detection of pleiotropy of QTNs is not as clear as for the marker SNPs. For instance, increased mutation rates decrease the pleiotropic FPR in the Gaussian but not the HoC regime. The influence of the equilibrium trait correlation also has an unclear effect on FPRs. This leads to rather similar FPRs for the Gaussian and HoC regimes (6–7%) despite large differences in genetic correlations (∼0% and ∼10% in the Gaussian and HoC regimes, respectively). In a previous version of this manuscript (Chebib and Guillaume 2020, version 4), we provided an analysis of the pleiotropic FPR when mapping nonpleiotropic QTN with different map distances between them. Our main finding was that the spurious pleiotropy detection rate was more influenced by the genetic correlation between the phenotypic traits than the map distance between the nonpleiotropic QTN, for only one combination of effect size and mutation rate (namely $\alpha =0.1,\;\mu =10^{-3}$, see Supplementary Table S5).

Although we have focused on characterizing spurious pleiotropy, as a large part of the literature on human GWAS does in order to avoid it, our results also show rather low DRs of true pleiotropy. The power to detect and measure true pleiotropy is especially low when trait architectures are more polygenic, as in the Gaussian regime where more loci of small effect are segregating. Whereas, when a single locus has large effects on multiple traits, it is more likely that a GWAS can detect true pleiotropy, which may then be used successfully to avoid possible undesired pleiotropic side-effects in targeted gene therapy (Li and Shen 2019). Another important factor is the statistical effect of taking a sample of the whole population, even though 1000 individuals already represent a well-sized sample in studies outside the human GWAS world. Had we taken a larger sample, we would have found a smaller number of false negatives (*e.g.*, see Supplementary Table S4). The salient consequence is that study design, threshold levels, and genetic correlations between traits will all affect the detection of genetic variants, whether the variants are causal themselves or linked to causal variants (Wagner and Zhang 2011; Hill and Zhang 2012a). The number of pleiotropic effects of a locus is likely under-represented by significance levels in association studies (Hill and Zhang 2012b).

### There is a difference between pleiotropy and linkage at the nucleotide level

Transgenic experiments and fine-scale association mapping may differentiate pleiotropy from linkage at the gene level (Mills *et al.* 2014; Archambeault *et al.* 2020). On the other hand, there is evidence that even in the same gene, adjacent polymorphisms affecting different traits in *Drosophila* can be in linkage equilibrium due to fine-scale recombination (Carbone *et al.* 2006; Flint and Mackay 2009). But, imagine a case where a mutation in a single base-pair has an effect on one trait and a mutation in the base-pair right next to the first base-pair has an effect on a second trait. Now imagine a second case where a mutation in a single base-pair has an effect on two traits. There still seems to be a distinction between these two cases because the probability of a change in both traits in the first case is the mutation rate squared compared to the second case where the probability of a change in both traits is just the mutation rate. Depending on the per-locus mutation rate this difference can be quite large (*e.g.*, $10^{-8}$*vs* $10^{-16}$). Even in this extreme case, there may indeed still be a gray area in the distinction between pleiotropy and linkage at a mutational level. Mutations may affect the pleiotropic degree (*e.g.*, like enzyme specificity) of a protein-coding gene and the degree to which the gene maintains multifunctionality may itself evolve (Guillaume and Otto 2012). If there is correlational selection between the catalytic functions of an enzyme, then some pleiotropic mutations that affect more than one catalytic ability will be favored, and genetic correlations will increase. With this in mind, it makes more sense from a theoretical and functional standpoint to refer to pleiotropy at the nucleotide level (or at the unit of a mutation), than at the gene or larger locus level (but this may depend on the questions of interest; Rockman 2012; Rausher and Delph 2015).

### Other factors

Even in the absence of correlational selection, it is possible to maintain genetic correlation through continued migration from a source population. High migration brings individuals whose combination of alleles will expand focal population variation in the direction of the source population. This corroborates previous results that showed that slow introgression of allelic combinations into a population can affect the genetic variance–covariance structure of that population (Guillaume and Whitlock 2007). Whether genetic covariance will be maintained in real populations depends on the nature of correlational selection on traits in the population of interest, since migration can reduce local fitness (*i.e.*, migration load) if allele combinations are not favored by selection or increase it if they are (Nosil *et al.* 2006; Bolnick and Otto 2013). Migration into a population will also affect FPRs since immigrating allele combinations will be in LD from the source population and will therefore increase the proportion of certain genotypes, even if there is no strong trait correlation in the source population. Although not investigated in this study, a structured population and/or a continual system of inbreeding in a population where there is correlational selection between polygenic traits can result in increased genetic covariation caused by larger LD (Lande 1984), which can in turn increase false-positive proportions.

## Conclusion

Pleiotropic loci maintain stronger genetic correlations between traits than linked loci affecting different traits even when no recombination occurs between the nonpleiotropic loci, and especially in the magnitude of empirically estimated mutation rates. Previous models of the maintenance of genetic covariation at mutation–selection equilibrium describe genetic correlations as a sole function of the strength of correlational selection, unless drift is taken into account. These models provide similar expectations for pleiotropic and tight linkage architectures. The discrepancy occurs because of the dependence of mutational covariance on the occurrence of mutations (and hence mutation the rate). Without high mutation rates, the ability to create genetic covariance between linked loci is low because there is a low probability of two simultaneous mutations with effects in the same direction. This low probability can be compensated by an increased number of linked loci in clustered architectures. Our results will have implications in the type of underlying architecture we expect to find in multitrait association studies. On the one hand, tighter linkage between causal loci maintains higher genetic correlations, leading to a greater proportion of false positives in pleiotropy tests. On the other hand, pleiotropic loci are overall more likely to be detected, especially when they affect tightly correlated and functionally distinct traits, increasing true DRs. In most cases though, it will remain challenging to distinguish between pleiotropy and linkage, both functionally and statistically. Our analyses suggest that a mix of multi- and univariate GWAA approaches can help weed out spurious pleiotropy.

## Data availability

The data for this study will be made available online through Zenodo online repository at https://zenodo.org/record/5469293 and code for simulations can be found at https://sourceforge.net/projects/nemo2/files/Publications-Code/ChebibGuillaume-PleiotropyOrLinkage-Genetics-2021/

Supplementary material is available at *GENETICS* online.

## Supplementary Material

iyab159_Supplementary_Figures_Tables

iyab159_Supplementary_Data

## References

[iyab159-B1] Archambeault SL , Bärtschi LR, Merminod AD, Peichel CL. 2020. Adaptation via pleiotropy and linkage: association mapping reveals a complex genetic architecture within the stickleback Eda locus. Evol Lett. 4:282–301. 32774879 10.1002/evl3.175 PMC7403726

[iyab159-B2] Arnold SJ. 1992. Constraints on phenotypic evolution. Am Nat. 140:S85–S107. 19426028 10.1086/285398

[iyab159-B3] Barghi N , Hermisson J, Schlötterer C. 2020. Polygenic adaptation: a unifying framework to understand positive selection. Nat Rev Genet. 21:769–781. 32601318 10.1038/s41576-020-0250-z

[iyab159-B4] Barrett RD , Hoekstra HE. 2011. Molecular spandrels: tests of adaptation at the genetic level. Nat Rev Genet. 12:767–780. 22005986 10.1038/nrg3015

[iyab159-B5] Benjamini Y , Hochberg Y. 1995. Controlling the false discovery rate: a practical and powerful approach to multiple testing. J R Statist Soc Ser B. 57:289–300.

[iyab159-B6] Bolnick DI , Otto SP. 2013. The magnitude of local adaptation under genotype-dependent dispersal. Ecol Evol. 3:4722–4735. 24363900 10.1002/ece3.850 PMC3867907

[iyab159-B7] Boyle EA , Li YI, Pritchard JK. 2017. An expanded view of complex traits: from polygenic to omnigenic. Cell. 169:1177–1186. 28622505 10.1016/j.cell.2017.05.038 PMC5536862

[iyab159-B8] Bullard DC , Ticknor C, Schimenti JC. 1992. Functional analysis of at complex responder locus transgene in mice. Mamm Genome. 3:579–587. 1421767 10.1007/BF00350625

[iyab159-B9] Bulmer M. 1972. The genetic variability of polygenic characters under optimizing selection, mutation and drift. Genet Res. 19:17–25. 5024710 10.1017/s0016672300014221

[iyab159-B10] Bulmer M. 1989. Maintenance of genetic-variability by mutation selection balance—a child’s guide through the jungle. Genome. 31:761–767.

[iyab159-B11] Bulmer MG . 1980. The Mathematical Theory of Quantitative Genetics. Oxford, UK: Clarendon Press.

[iyab159-B12] Bürger R. 2000. The mathematical theory of selection, recombination, and mutation. In: Wiley Series in Mathematical and Computational Biology, Ed. Levin S, Vol. 228. UK: Wiley & Sons, Ltd.

[iyab159-B13] Cai L , Fisher AL, Huang H, Xie Z. 2016. CRISPR-mediated genome editing and human diseases. Genes Dis. 3:244–251. 30258895 10.1016/j.gendis.2016.07.003 PMC6150104

[iyab159-B14] Cai Z , Dusza M, Guldbrandtsen B, Lund MS, Sahana G. 2020. Distinguishing pleiotropy from linked QTL between milk production traits and mastitis resistance in Nordic Holstein cattle. Genet Sel Evol. 52:1–15. 32264818 10.1186/s12711-020-00538-6 PMC7137482

[iyab159-B15] Carbone MA , Jordan KW, Lyman RF, Harbison ST, Leips J, et al 2006. Phenotypic variation and natural selection at catsup, a pleiotropic quantitative trait gene in Drosophila. Curr Biol. 16:912–919. 16682353 10.1016/j.cub.2006.03.051 PMC10766118

[iyab159-B16] Chantepie S , Chevin L-M. 2020. How does the strength of selection influence genetic correlations? Evol Lett. 4:468–478. 33312683 10.1002/evl3.201 PMC7719553

[iyab159-B17] Chebib J , Guillaume F. 2017. What affects the predictability of evolutionary constraints using a G-matrix? The relative effects of modular pleiotropy and mutational correlation. Evolution. 71:2298–2312. 28755417 10.1111/evo.13320

[iyab159-B18] Chebib J , Guillaume F. 2020. Pleiotropy or linkage? Their relative contributions to the genetic correlation of quantitative traits and detection by multi-trait GWA studies. bioRxiv 656413, doi: 10.1101/656413. PMC8664587 34849850

[iyab159-B19] Chesmore K , Bartlett J, Williams SM. 2018. The ubiquity of pleiotropy in human disease. Hum Genet. 137:39–44. 29164333 10.1007/s00439-017-1854-z

[iyab159-B20] Chung D , Yang C, Li C, Gelernter J, Zhao H. 2014. GPA: a statistical approach to prioritizing GWAS results by integrating pleiotropy and annotation. PLoS Genet. 10:e1004787. 25393678 10.1371/journal.pgen.1004787 PMC4230845

[iyab159-B21] Crow JF , Kimura M. 1964. The theory of genetic loads. In: Proceedings of the XI International Congress. Genetics, Vol. 2*,* p. 495–505. Oxford, UK: Pergamon Press.

[iyab159-B22] Draghi JA , Whitlock MC. 2012. Phenotypic plasticity facilitates mutational variance, genetic variance, and evolvability along the major axis of environmental variation. Evolution. 66:2891–2902. 22946810 10.1111/j.1558-5646.2012.01649.x

[iyab159-B23] Edelstein ML , Abedi MR, Wixon J. 2007. Gene therapy clinical trials worldwide to 2007—an update. J Gene Med. 9:833–842. 17721874 10.1002/jgm.1100

[iyab159-B24] Falconer DS , Mackay TF. 1996. Introduction to Quantitative Genetics, 4th ed. London: Longman.

[iyab159-B25] Fernandes SB , Zhang KS, Jamann TM, Lipka AE. 2021. How well can multivariate and univariate GWAS distinguish between true and spurious pleiotropy? Front Genet. 11:602526. 33584799 10.3389/fgene.2020.602526 PMC7873880

[iyab159-B26] Flint J , Mackay TF. 2009. Genetic architecture of quantitative traits in mice, flies, and humans. Genome Res. 19:723–733. 19411597 10.1101/gr.086660.108 PMC3647534

[iyab159-B27] Ford CB , Lin PL, Chase MR, Shah RR, Iartchouk O, et al 2011. Use of whole genome sequencing to estimate the mutation rate of mycobacterium tuberculosis during latent infection. Nat Genet. 43:482–486. 21516081 10.1038/ng.811 PMC3101871

[iyab159-B28] Ginn SL , Amaya AK, Alexander IE, Edelstein M, Abedi MR. 2018. Gene therapy clinical trials worldwide to 2017: an update. J Gene Med. 20:e3015. 29575374 10.1002/jgm.3015

[iyab159-B29] Gratten J , Visscher PM. 2016. Genetic pleiotropy in complex traits and diseases: implications for genomic medicine. Genome Med. 8:78. 27435222 10.1186/s13073-016-0332-x PMC4952057

[iyab159-B30] Griswold CK , Logsdon B, Gomulkiewicz R. 2007. Neutral evolution of multiple quantitative characters: a genealogical approach. Genetics. 176:455–466. 17339224 10.1534/genetics.106.069658 PMC1893077

[iyab159-B31] Guillaume F , Otto SP. 2012. Gene functional trade-offs and the evolution of pleiotropy. Genetics. 192:1389–1409. 22982578 10.1534/genetics.112.143214 PMC3512146

[iyab159-B32] Guillaume F , Rougemont J. 2006. Nemo: an evolutionary and population genetics programming framework. Bioinformatics. 22:2556–2557. 16882649 10.1093/bioinformatics/btl415

[iyab159-B33] Guillaume F , Whitlock MC. 2007. Effects of migration on the genetic covariance matrix. Evolution. 61:2398–2409. 17711463 10.1111/j.1558-5646.2007.00193.x

[iyab159-B34] Hastings A. 1989. Linkage disequilibrium and genetic variances under mutation-selection balance. Genetics. 121:857–860. 2721936 10.1093/genetics/121.4.857 PMC1203669

[iyab159-B35] Herrmann BG , Bauer H. 2012. The mouse t—haplotype: a selfish chromosome—genetics, molecular mechanism, and evolution. In: Evolution of the House Mouse, Ed. Macholan M, Baird SJE, Munclinger P, Pialek J. Vol. 3. p. 297. Cambridge University Press, Cambridge, UK.

[iyab159-B36] Hill WG , Zhang X-S. 2012a. Assessing pleiotropy and its evolutionary consequences: pleiotropy is not necessarily limited, nor need it hinder the evolution of complexity. Nat Rev Genet. 13:296. 22349131 10.1038/nrg2949-c1

[iyab159-B37] Hill WG , Zhang X-S. 2012b. On the pleiotropic structure of the genotype-phenotype map and the evolvability of complex organisms. Genetics. 190:1131–1137. 22214609 10.1534/genetics.111.135681 PMC3296247

[iyab159-B38] Hodgkin J. 1998. Seven types of pleiotropy. Int J Dev Biol. 42:501–505. 9654038

[iyab159-B39] Johnson T , Barton N. 2005. Theoretical models of selection and mutation on quantitative traits. Philos Trans R Soc Lond B Biol Sci. 360:1411–1425. 16048784 10.1098/rstb.2005.1667 PMC1569515

[iyab159-B40] Jones FC , Grabherr MG, Chan YF, Russell P, Mauceli E, et al; Broad Institute Genome Sequencing Platform & Whole Genome Assembly Team. 2012. The genomic basis of adaptive evolution in threespine sticklebacks. Nature. 484:55–61. 22481358 10.1038/nature10944 PMC3322419

[iyab159-B41] Jordan DM , Verbanck M, Do R. 2019. HOPS: a quantitative score reveals pervasive horizontal pleiotropy in human genetic variation is driven by extreme polygenicity of human traits and diseases. Genome Biol. 20:222. 31653226 10.1186/s13059-019-1844-7 PMC6815001

[iyab159-B42] Keightley PD , Pinharanda A, Ness RW, Simpson F, Dasmahapatra KK, et al 2015. Estimation of the spontaneous mutation rate in *Heliconius melpomene*. Mol Biol Evol. 32:239–243. 25371432 10.1093/molbev/msu302 PMC4271535

[iyab159-B43] Lande R. 1975. The maintenance of genetic variability by mutation in a polygenic character with linked loci. Genet Res. 26:221–235. 1225762 10.1017/s0016672300016037

[iyab159-B44] Lande R. 1980. The genetic covariance between characters maintained by pleiotropic mutations. Genetics. 94:203–215. 17248993 10.1093/genetics/94.1.203 PMC1214134

[iyab159-B45] Lande R. 1984. The genetic correlation between characters maintained by selection, linkage and inbreeding. Genet Res. 44:309–320. 6530140 10.1017/s0016672300026549

[iyab159-B46] Latter B. 1960. Natural selection for an intermediate optimum. Aust J Biol Sci. 13:30–35.

[iyab159-B47] Li T , Shen X. 2019. Pleiotropy complicates human gene editing: Ccr5*δ*32 and beyond. Front Genet. 10:669. 31417603 10.3389/fgene.2019.00669 PMC6684159

[iyab159-B48] Lindsay SJ , Rahbari R, Kaplanis J, Keane T, Hurles ME. 2019. Similarities and differences in patterns of germline mutation between mice and humans. Nat Commun. 10:1–12. 31492841 10.1038/s41467-019-12023-w PMC6731245

[iyab159-B49] Liu M , Jiang Y, Wedow R, Li Y, Brazel DM, et al 2019. Association studies of up to 1.2 million individuals yield new insights into the genetic etiology of tobacco and alcohol use. Nat Genet. 51:237–244. 30643251 10.1038/s41588-018-0307-5 PMC6358542

[iyab159-B50] Lynch M , Walsh B, et al 1998. Genetics and Analysis of Quantitative Traits, Vol. 1. MA: Sinauer Associates, Sunderland, Massachussetts.

[iyab159-B51] Mackay TFC. 2001. The genetic architecture of quantitative traits. Annu Rev Genet. 35:303–339. 11700286 10.1146/annurev.genet.35.102401.090633

[iyab159-B52] Mills MG , Greenwood AK, Peichel CL. 2014. Pleiotropic effects of a single gene on skeletal development and sensory system patterning in sticklebacks. Evodevo. 5:5. 24499504 10.1186/2041-9139-5-5 PMC3976036

[iyab159-B53] Myers S , Spencer C, Auton A, Bottolo L, Freeman C, et al 2006. The distribution and causes of meiotic recombination in the human genome. Biochem Soc Trans. 34:526–530. 16856851 10.1042/BST0340526

[iyab159-B54] Nachman MW , Crowell SL. 2000. Estimate of the mutation rate per nucleotide in humans. Genetics. 156:297–304. 10978293 10.1093/genetics/156.1.297 PMC1461236

[iyab159-B55] Nosil P , Crespi B, Sandoval C, Kirkpatrick M. 2006. Migration and the genetic covariance between habitat preference and performance. Am Nat. 167:E66–E78. 16673338 10.1086/499383

[iyab159-B56] Parkes M , Cortes A, Van Heel DA, Brown MA. 2013. Genetic insights into common pathways and complex relationships among immune-mediated diseases. Nat Rev Genet. 14:661–673. 23917628 10.1038/nrg3502

[iyab159-B57] Peichel CL , Marques DA. 2017. The genetic and molecular architecture of phenotypic diversity in sticklebacks. Phil Trans R Soc B. 372:20150486. 27994127 10.1098/rstb.2015.0486 PMC5182418

[iyab159-B58] Pickrell JK , Berisa T, Liu JZ, Ségurel L, Tung JY, et al 2016. Detection and interpretation of shared genetic influences on 42 human traits. Nat Genet. 48:709–717. 27182965 10.1038/ng.3570 PMC5207801

[iyab159-B59] Platt A , Vilhjálmsson BJ, Nordborg M. 2010. Conditions under which genome-wide association studies will be positively misleading. Genetics. 186:1045–1052. 20813880 10.1534/genetics.110.121665 PMC2975277

[iyab159-B60] Rausher MD , Delph LF. 2015. Commentary: when does understanding phenotypic evolution require identification of the underlying genes? Evolution. 69:1655–1664. 25973520 10.1111/evo.12687

[iyab159-B61] Rockman MV. 2012. The QTN program and the alleles that matter for evolution: all that’s gold does not glitter. Evolution. 66:1–17. 22220860 10.1111/j.1558-5646.2011.01486.x PMC3386609

[iyab159-B62] Saltz JB , Hessel FC, Kelly MW. 2017. Trait correlations in the genomics era. Trends Ecol Evol. 32:279–290. 28139251 10.1016/j.tree.2016.12.008

[iyab159-B63] Sella G , Barton NH. 2019. Thinking about the evolution of complex traits in the era of genome-wide association studies. Annu Rev Genomics Hum Genet. 20:461–493. 31283361 10.1146/annurev-genom-083115-022316

[iyab159-B64] Shikov AE , Skitchenko RK, Predeus AV, Barbitoff YA. 2020. Phenome-wide functional dissection of pleiotropic effects highlights key molecular pathways for human complex traits. Sci Rep. 10:1037. 31974475 10.1038/s41598-020-58040-4 PMC6978431

[iyab159-B65] Stearns FW. 2010. One hundred years of pleiotropy: a retrospective. Genetics. 186:767–773. 21062962 10.1534/genetics.110.122549 PMC2975297

[iyab159-B66] Storey JD , Bass AJ, Dabney A, Robinson D. 2019. qvalue: Q-value estimation for false discovery rate control. R package version 2.14.1., http://github.com/jdstorey/qvalue.

[iyab159-B67] Storey JD , Tibshirani R. 2003. Statistical significance for genomewide studies. Proc Natl Acad Sci U S A. 100:9440–9445. 12883005 10.1073/pnas.1530509100 PMC170937

[iyab159-B68] Turelli M , Barton NH. 1990. Dynamics of polygenic characters under selection. Theor Popul Biol. 38:1–57.

[iyab159-B69] Turelli M. 1984. Heritable genetic variation via mutation-selection balance: Lerch’s zeta meets the abdominal bristle. Theor Popul Biol. 25:138–193. 6729751 10.1016/0040-5809(84)90017-0

[iyab159-B70] Turelli M. 1985. Effects of pleiotropy on predictions concerning mutation-selection balance for polygenic traits. Genetics. 111:165–195. 4029610 10.1093/genetics/111.1.165 PMC1202595

[iyab159-B71] Verbanck M , Chen C-Y, Neale B, Do R. 2018. Detection of widespread horizontal pleiotropy in causal relationships inferred from Mendelian randomization between complex traits and diseases. Nat Genet. 50:693–698. 29686387 10.1038/s41588-018-0099-7 PMC6083837

[iyab159-B72] Visscher PM , Wray NR, Zhang Q, Sklar P, McCarthy MI, et al 2017. 10 years of GWAS discovery: biology, function, and translation. Am J Hum Genet. 101:5–22. 28686856 10.1016/j.ajhg.2017.06.005 PMC5501872

[iyab159-B73] Visscher PM , Yang J. 2016. A plethora of pleiotropy across complex traits. Nat Genet. 48:707–708. 27350602 10.1038/ng.3604

[iyab159-B74] Wagner GP , Zhang J. 2011. The pleiotropic structure of the genotype–phenotype map: the evolvability of complex organisms. Nat Rev Genet. 12:204–213. 21331091 10.1038/nrg2949

[iyab159-B75] Walsh B , Blows MW. 2009. Abundant genetic variation + strong selection = multivariate genetic constraints: a geometric view of adaptation. Annu Rev Ecol Evol Syst. 40:41–59.

[iyab159-B76] Walsh B , Lynch M. 2018. Evolution and Selection of Quantitative Traits. Clarendon St., London, UK: Sinauer Associates, Oxford University Press.

[iyab159-B77] Watanabe K , Stringer S, Frei O, Mirkov MU, Leeuw C. D, et al 2019. A global overview of pleiotropy and genetic architecture in complex traits. Nat Genet. 51:1339–1348. 31427789 10.1038/s41588-019-0481-0

[iyab159-B78] Wei W-H , Hemani G, Haley CS. 2014. Detecting epistasis in human complex traits. Nat Rev Genet. 15:722–733. 25200660 10.1038/nrg3747

[iyab159-B79] Wei X , Nielsen R. 2019. Ccr5-*δ* 32 is deleterious in the homozygous state in humans. Nat Med. 25:909–910. 31160814 10.1038/s41591-019-0459-6 PMC6613792

[iyab159-B80] Wright S. 1977. Evolution and the genetics of populations. Vol. 3. Chicago: University of Chicago Press.

[iyab159-B81] Zhang XS , Hill WG. 2003. Multivariate stabilizing selection and pleiotropy in the maintenance of quantitative genetic variation. Evolution. 57:1761–1775. 14503618 10.1111/j.0014-3820.2003.tb00584.x

[iyab159-B82] Zhou X , Stephens M. 2014. Efficient multivariate linear mixed model algorithms for genome-wide association studies. Nat Methods. 11:407–409. 24531419 10.1038/nmeth.2848 PMC4211878

[iyab159-B83] Zhu Z , Zhang F, Hu H, Bakshi A, Robinson MR, et al 2016. Integration of summary data from GWAS and eQTL studies predicts complex trait gene targets. Nat Genet. 48:481–487. 27019110 10.1038/ng.3538

