# Supplementary material for: Pleiotropy or linkage? Their relative contributions to the genetic correlation of quantitative traits and detection by multitrait GWA studies
Source: Genetics. 2021 Sep 25;219(4):iyab159. doi: 10.1093/genetics/iyab159 (PMC8664587; doi:10.1093/genetics/iyab159)
Supplement: iyab159_Supplementary_Figures_Tables [file iyab159_supplementary_figures_tables.pdf]

Supplemental information for: Pleiotropy or linkage? Their relative contributions to the genetic correlation of quantitative traits and detection by multi-trait GWA studies.

Jobran Chebib*,† and Frédéric Guillaume*,‡

*Department of Evolutionary Biology and Environmental Studies, University of Zürich, Winterthurerstr. 190, 8057 Zürich, Switzerland

†Institute of Evolutionary Biology, University of Edinburgh, Edinburgh, EH9 3FL, UK

‡Organismal and Evolutionary Biology Research Program, University of Helsinki, PL 65, 00014 Helsinki, Finland

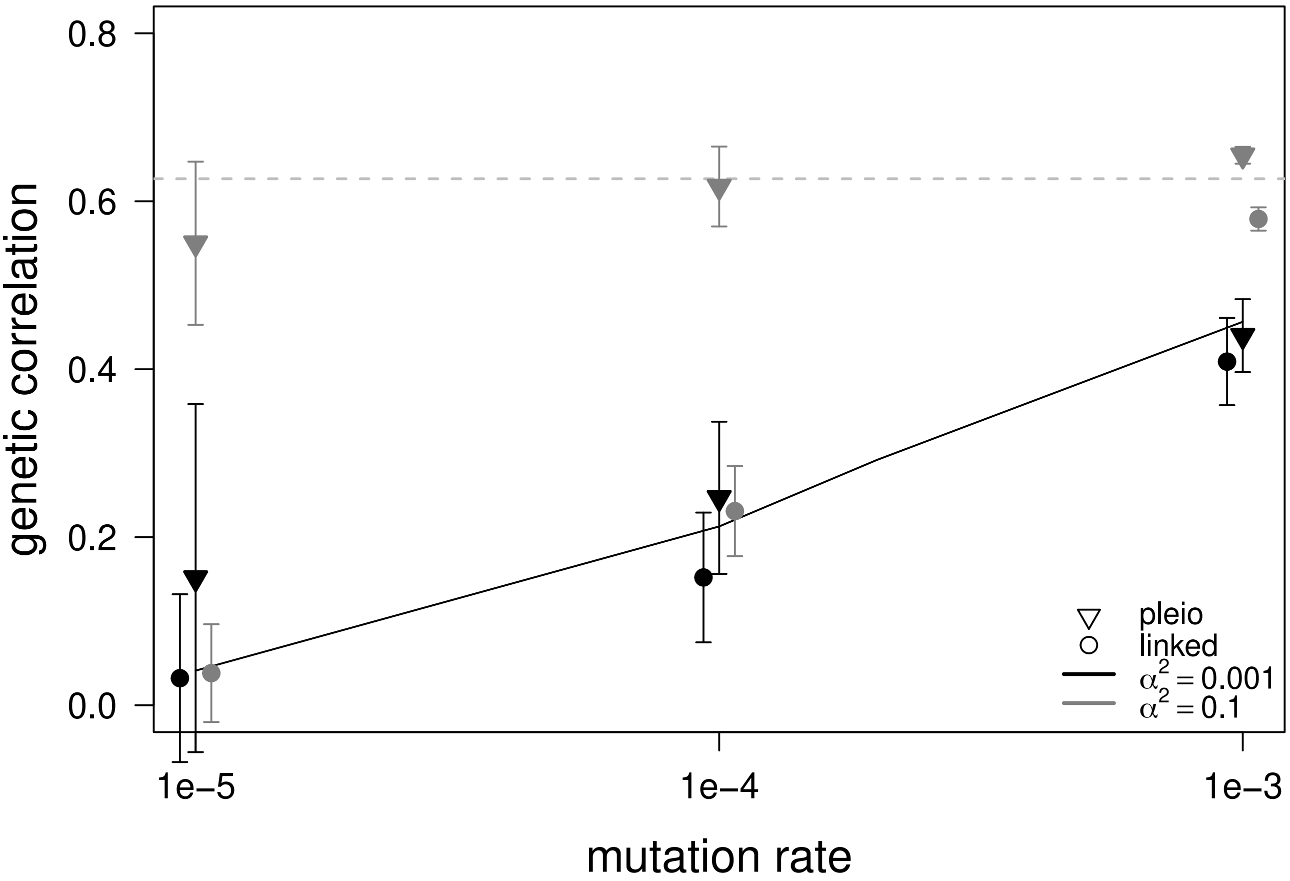

Figure S1: Equilibrium genetic correlation under the Gaussian regime (black symbols) and the house-of-cards regime (dark gray symbols) for 60 independent pairs of completely linked non-pleiotropic loci (circles) or 60 independent pleiotropic loci (triangles) after 50,000 generations of stabilizing selection. The continuous line shows the genetic correlation at mutation-selection-drift balance predicted by equation (9) in Chantepie and Chevin (2020). The dashed horizontal gray line represents the mutation-invariant maximum genetic correlation predicted by equation (3) in the main text. Parameters are: $N = 5000$, $\omega^2 = 100$, $\rho_\omega = 0.9$. In black $\alpha^2 = 0.001$, and $\mu = 10^{-3}$ (Gaussian regime). In dark gray $\alpha^2 = 0.1$, and $\mu = 10^{-5}$ (house-of-cards regime).

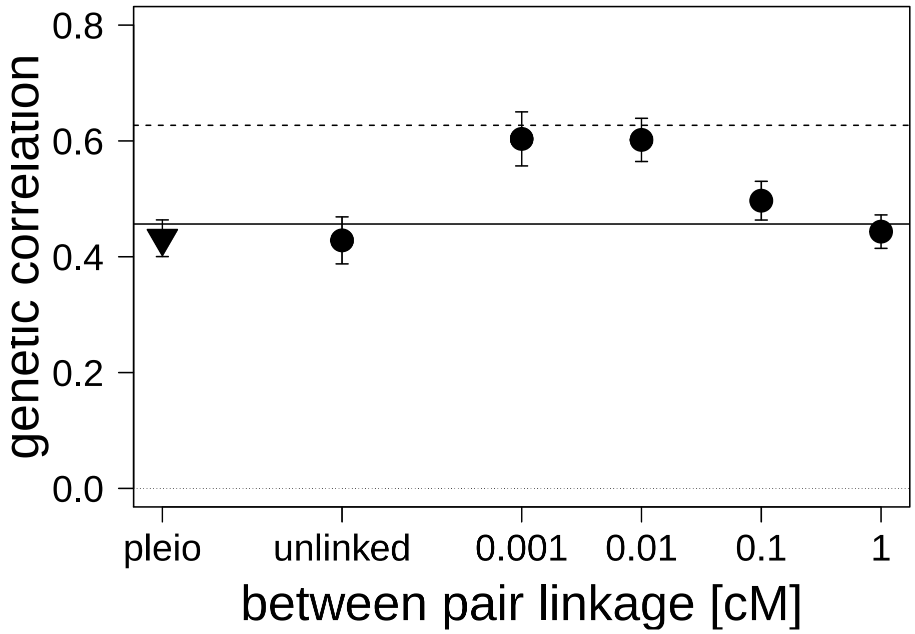

Figure S2: Equilibrium genetic correlation for loci in the Gaussian regime and between-pair linkage. Error bars correspond to one standard deviation over 30 replicates. $N = 5000$, $\omega^2 = 100$, $\rho_\omega = 0.9$, $\alpha^2 = 0.001$, and $\mu = 10^{-3}$. The 120 pairs of non-pleiotropic loci were placed on a single chromosome at map distance shown on the x-axis. Pleiotropic loci are depicted with an inverse triangle. The solid line represents Chantepie and Chevin (2020) mutation-selection-drift expectation. The dashed line represents Lande's (1984) expectation.

Table S1: Statistics of the distribution of allelic effects at linked non-pleiotropic QTL at mutation-selection-drift equilibrium. Values are averages and SEs for 30 replicates. The genic covariance (cov_{genic}) is the within gamete covariance of allelic effects affecting trait 1 and trait 2 within a pair of two non-pleiotropic linked loci, summed over all pairs and the two gametes. The genotypic covariance (cov_{genot}) is the covariance of the genotypic values of trait 1 and 2. It thus includes all contributions of pairwise genic covariance, within and between pairs, and within and between gametes. The genic variance is the sum of the additive variance of each QTL. The genotypic variance it the variance of the genotypic values. The r^2 is the multi-allelic within-gamete LD within pairs of linked QTL. The number of alleles is the mean number of unique real values segregating at each locus. The number of QTL is the number of segregating QTL in the population. All statistics are computed over all individuals in the population. There is free recombination between the pairs of linked loci. Symbols are: n number of loci/pairs of loci, α^2 mutation variance (effect size), μ mutation rate. Note that although the between-pair covariance is not included in the genic covariance shown here, the genic and genotypic covariance are close.

n	α^2	μ	var_{genot}	cov_{genot}	var_{genic}	cov_{genic}	r^2	kurtosis	#alleles	#QTL
120	0.001	1e-05	0.017 (0.001)	0.001 (0.000)	0.035 (0.001)	0.001 (0.000)	0.003 (0.001)	467.46 (20.84)	2.69 (0.02)	202.5 (1.1)
		1e-04	0.159 (0.004)	0.027 (0.002)	0.320 (0.007)	0.025 (0.002)	0.013 (0.003)	3.84 (0.23)	16.15 (0.07)	240.0 (0.0)
		1e-03	1.050 (0.012)	0.451 (0.015)	2.192 (0.022)	0.424 (0.012)	0.025 (0.004)	0.09 (0.02)	111.98 (0.15)	240.0 (0.0)
120	0.1	1e-05	0.085 (0.003)	0.002 (0.001)	0.172 (0.006)	0.001 (0.000)	0.001 (0.000)	1004.34 (48.31)	2.15 (0.02)	174.5 (1.9)
		1e-04	1.089 (0.020)	0.273 (0.011)	2.346 (0.044)	0.217 (0.012)	0.014 (0.002)	38.06 (1.13)	12.86 (0.05)	240.0 (0.0)
		1e-03	12.256 (0.084)	7.821 (0.090)	36.308 (0.227)	6.613 (0.111)	0.026 (0.002)	1.11 (0.02)	106.52 (0.22)	240.0 (0.0)
60	0.001	1e-05	0.009 (0.001)	0.000 (0.000)	0.019 (0.001)	0.000 (0.000)	-0.000 (0.002)	470.47 (35.07)	2.72 (0.02)	100.6 (1.0)
		1e-04	0.079 (0.003)	0.012 (0.002)	0.158 (0.005)	0.012 (0.002)	0.018 (0.004)	4.42 (0.84)	16.09 (0.08)	120.0 (0.0)
		1e-03	0.532 (0.009)	0.218 (0.010)	1.087 (0.019)	0.214 (0.010)	0.025 (0.004)	0.12 (0.04)	111.95 (0.33)	120.0 (0.0)
60	0.1	1e-05	0.044 (0.002)	0.002 (0.001)	0.088 (0.003)	0.002 (0.001)	0.001 (0.001)	995.36 (69.30)	2.16 (0.02)	87.9 (1.1)
		1e-04	0.524 (0.013)	0.122 (0.010)	1.089 (0.028)	0.105 (0.010)	0.027 (0.004)	48.81 (2.03)	12.59 (0.09)	120.0 (0.0)
		1e-03	5.862 (0.068)	3.395 (0.057)	14.362 (0.156)	3.096 (0.057)	0.050 (0.004)	1.42 (0.04)	104.65 (0.18)	120.0 (0.0)

Table S2: Comparison of genic and genotypic covariance between linked loci (0cM) and pleiotropic loci. Values are averages over 30 replicates. n number of loci/pairs of loci, α^2 mutation variance (effect size), μ mutation rate. Same comments and parameter values as in Table S1.

			linked loci		pleiotropic loci	
n	α^2	μ	cov_{genic}	cov_{genot}	cov_{genic}	cov_{genot}
120	0.001	1e-05	0.0008	0.0007	0.003	0.003
		1e-04	0.025	0.027	0.036	0.037
		1e-03	0.424	0.451	0.419	0.443
120	0.1	1e-05	0.0012	0.0023	0.070	0.073
		1e-04	0.217	0.273	0.741	0.727
		1e-03	6.613	7.821	7.867	7.909
60	0.001	1e-05	0.0003	0.0004	0.0015	0.0015
		1e-04	0.012	0.0124	0.021	0.0202
		1e-03	0.214	0.218	0.227	0.235
60	0.1	1e-05	0.0015	0.0017	0.024	0.027
		1e-04	0.105	0.122	0.337	0.334
		1e-03	3.096	3.395	3.605	3.554

Table S3: Genic and genotypic covariance in the case of fully linked (0cM) pairs non-pleiotropic loci with reduction of recombination between pairs. Simulation parameters are otherwise similar to Table S1 for 120 pairs of non-pleiotropic loci. Contrary to Table S1, the genic covariance is calculated on all between-pair associations, between loci affecting trait 1 in one pair and trait 2 in another pair, over all possible pairs. It also includes the within-pair associations. The genotypic covariance is calculated as in Table S1. Note that although the covariance increases with the map distance between pairs (cM in first column), the resulting genetic correlation decreases because of the concomitant increase in the genotypic variance.

		$\alpha^2 = 0.001$ (Gauss)		$\alpha^2 = 0.1$ (HoC)	
cM	μ	cov_{genic}	cov_{genot}	cov_{genic}	cov_{genot}
0.001	1e-05	0.003	0.004	0.057	0.061
	1e-04	0.038	0.040	0.343	0.342
	1e-03	0.125	0.135	1.129	1.136
0.01	1e-05	0.003	0.002	0.078	0.075
	1e-04	0.046	0.046	0.663	0.666
	1e-03	0.281	0.266	1.728	1.697
0.1	1e-05	0.001	0.001	0.015	0.015
	1e-04	0.039	0.039	1.383	1.370
	1e-03	0.438	0.436	5.649	5.612
1.0	1e-05	0.0003	0.0004	0.003	0.005
	1e-04	0.028	0.028	0.459	0.486
	1e-03	0.451	0.460	8.956	9.054

Table S4: [same as Table 3 in the main text but with sample size = 5000] Summary of the univariate analyses of causative QTN associations with the two traits for linked pairs of non-pleiotropic and pleiotropic bi-allelic QTL. The pleiotropy false positive rate or spurious pleiotropy (FPR) is the proportion of non-pleiotropic QTNs significantly associated with both traits in two independent univariate GWAA. The false negative rate (FNR) is the proportion of segregating pleiotropic QTN not associated with the two traits in two independent GWAA. The mean r^2 measures LD within pairs of non-pleiotropic QTL bearing at least one significant QTN. The GWAA and the genetic correlation (G_{cor}) are computed on the whole population of 5000 individuals. The strength of correlational selection is $\rho_\omega = 0.9$ unless specified otherwise. Other simulation parameters are as presented before.

							QTN	
	α^2	μ		r^2	G_{cor}	N_{sign}	N_{pleio}	N_{segr}
Linkage			*FPR*					
Gauss.	0.001	1e-03	0.162 (0.004)	0.019 (0.001)	-4e-04 (0.003)	240.0 (0.0)	38.9 (0.9)	240.0 (0.0)
-	0.1	1e-03	0.330 (0.007)	0.022 (0.000)	0.206 (0.003)	240.0 (0.0)	79.3 (1.6)	240.0 (0.0)
-	0.001	1e-05	0.283 (0.006)	0.224 (0.010)	0.002 (0.008)	154.9 (1.1)	44.0 (1.1)	203.0 (5.1)
HoC	0.1	1e-05	0.118 (0.005)	0.138 (0.019)	0.100 (0.015)	127.6 (1.2)	15.0 (0.6)	175.5 (8.0)
Pleiotropy			*FNR*					
Gauss.	0.001	1e-03	0.017 (0.002)	− (–)	-3e-04 (0.003)	120.0 (0.0)	118.0 (0.2)	120.0 (0.0)
-$^{(a)}$	0.1	1e-03	0.007 (0.001)	− (–)	0.189 (0.003)	120.0 (0.0)	119.1 (0.1)	120.0 (0.0)
-$^{(b)}$	0.001	1e-05	0.613 (0.008)	− (–)	0.219 (0.013)	90.9 (0.7)	42.7 (0.9)	110.4 (2.9)
HoC$^{(b)}$	0.1	1e-05	0.669 (0.007)	− (–)	0.230 (0.012)	85.5 (0.9)	35.8 (0.8)	108.1 (3.0)

$^{(a)}$ $\rho_\omega = 0.85$; $^{(b)}$ $\rho_\omega = 0.01$

Table S5: Reproduced from Chebib and Guillaume (2020). Results of GWA analyses for different architectures with average false negatives (Type II errors) for pleiotropic architectures and false positives (Type I errors) for linkage architectures, as well as linkage disequilibrium (LD) measurement averages for short-distance (physically linked loci) and long-distance (unlinked loci) comparisons. The genetic architectures in the bottom half of the table have higher genetic correlations than the top half (created by adjusting correlational selection) to compare the differences at different genetic correlation. See Chebib and Guillaume (2020) for details of the simulations.

Genetic Architecture	Genetic Cor (SE)	Type I/II Error %	D' short	D' long	R^2 short	R^2 long
Pleiotropy	0.308 (0.0046)	0.35%	NA	0.018	NA	0.00027
Linkage (0cM)	0.300 (0.0055)	22.06%	0.37	0.023	0.089	0.00026
Linkage (0.1cM)	0.300 (0.0045)	20.17%	0.26	0.025	0.047	0.00027
Linkage (1cM)	0.308 (0.0035)	18.28%	0.13	0.030	0.007	0.00027
Pleiotropy	0.407 (0.0048)	0.32%	NA	0.018	NA	0.00027
Linkage (0cM)	0.398 (0.0074)	28.76%	0.43	0.025	0.107	0.00027
Linkage (0.1cM)	0.408 (0.0035)	28.46%	0.30	0.027	0.050	0.00027
Linkage (1cM)	0.404 (0.0029)	25.34%	0.19	0.048	0.006	0.00027

References

Chantepie, S. and Chevin, L.-M. (2020). How does the strength of selection influence genetic correlations? *Evolution Letters*, 4(6):468–478.

Chebib, J. and Guillaume, F. (2020). Pleiotropy or linkage? Their relative contributions to the genetic correlation of quantitative traits and detection by multi-trait GWA studies. *bioRxiv*, page 656413.
